# Induction of human pluripotent stem cells into kidney tissues by synthetic mRNAs encoding transcription factors

**DOI:** 10.1038/s41598-018-37485-8

**Published:** 2019-01-29

**Authors:** Ken Hiratsuka, Toshiaki Monkawa, Tomohiko Akiyama, Yuhki Nakatake, Mayumi Oda, Sravan Kumar Goparaju, Hiromi Kimura, Nana Chikazawa-Nohtomi, Saeko Sato, Keiichiro Ishiguro, Shintaro Yamaguchi, Sayuri Suzuki, Ryuji Morizane, Shigeru B. H. Ko, Hiroshi Itoh, Minoru S. H. Ko

**Affiliations:** 1 Department of Systems Medicine, Keio University School of Medicine, 35 Shinanomachi, Shinjuku, Tokyo, 160-8582 Japan; 2 Department of Nephrology, Endocrinology, and Metabolism, Keio University School of Medicine, 35 Shinanomachi, Shinjuku, Tokyo, 160-8582 Japan; 3 Medical Education Center, Keio University School of Medicine, 35 Shinanomachi, Shinjuku, Tokyo, 160-8582 Japan; 4 Institute of Molecular Embryology and Genetics, Kumamoto University, 2-2-1 Honjo, Chuo-ku, Kumamoto, 860-0811 Japan; 5 Renal Division, Department of Medicine, Brigham and Women’s Hospital, Boston, Massachusetts, USA; 6 Department of Medicine, Harvard Medical School, Boston, Massachusetts, USA; 7 Harvard Stem Cell Institute, Cambridge, Massachusetts, USA

## Abstract

The derivation of kidney tissues from human pluripotent stem cells (hPSCs) and its application for replacement therapy in end-stage renal disease have been widely discussed. Here we report that consecutive transfections of two sets of synthetic mRNAs encoding transcription factors can induce rapid and efficient differentiation of hPSCs into kidney tissues, termed induced nephron-like organoids (iNephLOs). The first set - FIGLA, PITX2, ASCL1 and TFAP2C, differentiated hPSCs into SIX2^+^SALL1^+^ nephron progenitor cells with 92% efficiency within 2 days. Subsequently, the second set - HNF1A, GATA3, GATA1 and EMX2, differentiated these cells into PAX8^+^LHX1^+^ pretubular aggregates in another 2 days. Further culture in both 2-dimensional and 3-dimensional conditions produced iNephLOs containing cells characterized as podocytes, proximal tubules, and distal tubules in an additional 10 days. Global gene expression profiles showed similarities between iNephLOs and the human adult kidney, suggesting possible uses of iNephLOs as *in vitro* models for kidneys.

## Introduction

Chronic kidney disease is a global health issue with increasing numbers of end-stage renal disease patients who require renal replacement therapy (RRT)^1,2^. Once patients start RRT, recovery of renal function is difficult, and the progression of dialysis-related complications leads to a reduced quality of life. Derivation of kidney cells, tissues, and organs from human pluripotent stem cells (hPSCs) such as embryonic stem cells (hESCs) and induced pluripotent stem cells (hiPSCs), and their transplantation into patients as therapeutic interventions have been widely discussed as methods to potentially restore kidney function^3–6^.

As a first step, several *in vitro* differentiation methods, such as directed differentiation from hESCs and hiPSCs, and direct conversion from terminally differentiated cells to renal lineages have been reported^7–13^. Current protocols for directed differentiation using growth factors and chemical compounds usually involve multi-step procedures of changes of cell culture media, which lead to the generation of kidney organoids containing multiple nephron-like segments^7,10,11^. It is known that these methods show varied differentiation efficiency between different hPSC cell lines based on patient-specific genetic background^14^ or epigenetic status^15,16^. Alternatively, direct reprograming methods using transcription factor (TF) expression vectors (viral and plasmid) have also been developed, which lead to the generation of renal lineage cell types^12,13^. However, because of possible genome modification by viruses and plasmids, these procedures may not be suitable for clinical applications. Furthermore, only limited renal cell types have been generated by these methods. Recently, we have demonstrated that synthetic mRNAs can be transfected efficiently (>90%) in hPSCs^17,18^. We have also reported that synthetic mRNAs encoding TFs can differentiate hPSCs towards neurons, myocytes, and lacrimal gland epithelial-like cells^17–20^. Due to its non-mutagenic feature, this synthetic mRNA-based technology may be suitable for possible future clinical applications. We also reasoned that the transient nature of TF expression by synthetic mRNA-based technology enables activation of multiple TFs in a sequential manner, which may help to obtain cells at different stages of renal development and heterogeneous multi-segmented renal cells.

In this study, we initially attempted to induce hPSCs directly into renal tubular cells expressing cadherin 16 (CDH16: also known as kidney-specific protein, KSP), which is expressed in all tubular segments of nephrons with higher expression in distal segments^21,22^ and was used to identify renal tubular cells during the differentiation of mouse and human ES cells^23,24^. However, our initial efforts resulted in the generation of only partially differentiated kidney tubular cells. We, therefore, formulated a strategy to generate kidney tissues through nephron progenitor cells (NPCs) and identified two different sets of four TFs: the first set (FIGLA, PITX2, ASCL1 and TFAP2C) to induce NPCs from hPSCs; the second set (HNF1A, GATA3, GATA1 and EMX2) to induce nephron epithelial cells from the NPCs. Combined with three-dimensional suspension culture, the sequential administration of these TFs successfully generated, in 14 days, kidney tissues containing structures with characteristics of proximal and distal renal tubules, and glomeruli.

## Results

### Identification of key TFs for induction of renal lineages

**Figure 1:**
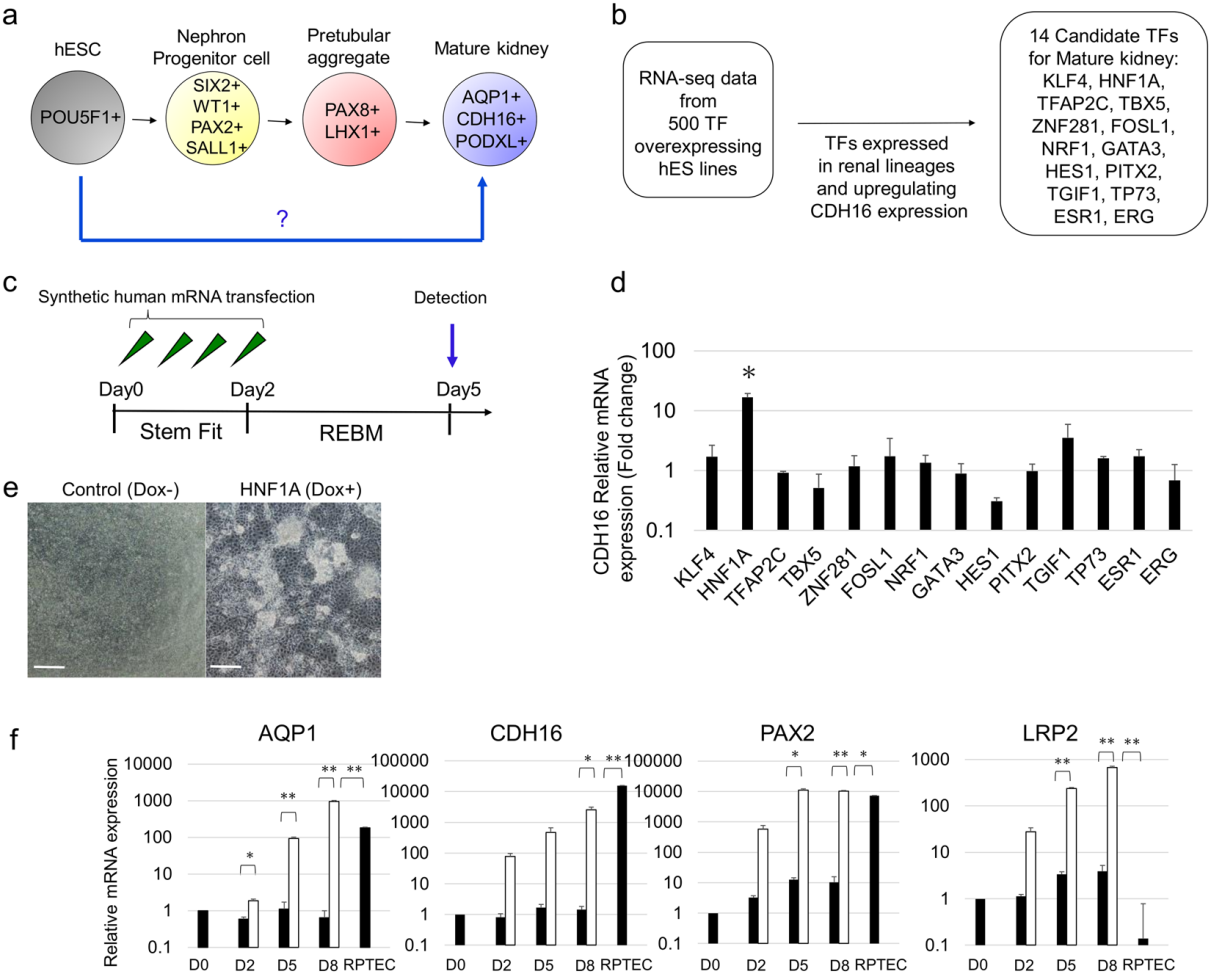
Identification of a renal-lineage master regulatory TF. (**a**) Diagram of differentiation into mature kidney. (**b**) Flowchart of an approach to identify renal linage master regulatory TFs. (**c**) Schematic representation of time course of mRNA transfection of candidate TFs. (**d**) Relative mRNA expression levels of CDH16 after mRNA transfection of candidate TFs into hESCs. (**e**) Representative bright-field images on day 5 of differentiation. Scale bar, 100 μm. (**f**) Time course of gene expression of AQP1, CDH16, PAX2, and LRP2 in transgene HNF1A-induced hESCs (white bars), in transgene un-induced hESCs, and in RPTECs (black bars) by qRT-PCR from day 0 to 8 n = 2. Data represent mean + s.e.m. P-values were determined by a Student’s t-test. *P < 0.05; **P < 0.01.

To identify key TFs that can facilitate the differentiation of hPSCs into kidney lineage cells, we used our human gene expression correlation matrix (manuscript in preparation), which was generated essentially in the same manner as the mouse gene expression correlation matrix^25–27^. Among approximately 500 TFs included in the matrix, we chose 66 top ranked TFs, whose overexpression shifted the transcriptome of hPSCs toward kidney lineage cells. We further reduced the number of TFs to 14 based on their ability to induce the expression of CDH16 – a renal tubule specific marker (Fig. 1a,b). We generated synthetic mRNAs for each of the 14 TFs and transfected them individually into hESCs (Fig. 1c). We found that by day 5, a synthetic mRNA encoding HNF1A (syn-HNF1A) induced CDH16 expression in hESCs by 10 times higher than the other 13 TFs as measured by quantitative RT-PCR (qRT-PCR) (Fig. 1d). To validate the effect of syn-HNF1A on CDH16 expression, we established a hESC line, wherein HNF1A expression can be induced by the addition of doxycycline (Dox) to the cell culture medium (Supplementary Fig. 1a). When HNF1A was induced, the cells began to form dense epithelial clusters (Fig. 1e). The induction of HNF1A was confirmed by qRT-PCR analysis (Supplementary Fig. 1b) as well as Western blotting and immunostaining (Supplementary Fig. 1c,d). We found that the overexpression of HNF1A upregulated the proximal tubule-related markers AQP1, CDH16, PAX2, and LRP2 (also known as MEGALIN) (Fig. 1f)^28,29^. The expression levels of these genes, except for CDH16, were comparable or even higher compared to those in primary culture of human renal proximal tubular epithelial cells (RPTECs). Immunostaining also revealed the presence of those proximal tubule-related markers (Supplementary Fig. 1e). Therefore, we decided to use HNF1A as a master regulatory TF towards renal lineage differentiation. However, in a recent study for direct differentiation of hESC into kidney lineage, HNF4A and HNF1B were reported as the master regulatory TFs for induction of CDH16^13^. In their study, HNF4A and HNF1B were implicated in nephrogenesis based on *in silico* mouse and xenopus expression arrays. We hypothesized that the master regulatory TF is species-specific and might be different between human and mouse. To test this hypothesis, we transfected hESCs with each of the synthetic mRNAs encoding for HNF4A, HNF1B, and HNF1A, and found that only HNF1A, but not HNF4A or HNF1B, upregulated the CDH16 mRNA level by Day 5 (Supplementary Fig. 1f). These results suggest the presence of species specificity for the involvement of TFs in hPSC differentiation.

In a previous study, we demonstrated that silencing POU5F1 (also known as OCT4, OCT3/4) accelerated MYOD1-mediated myogenic differentiation of hPSCs^17^. To test whether suppressing POU5F1 expression is also beneficial for kidney differentiation, we transfected siRNA targeting POU5F1 (siPOU5F1) with syn-HNF1A and determined the efficiency of differentiation. We found that the addition of siPOU5F1 increased the expression of renal lineage markers as measured by qRT-PCR, whereas the expression of the pluripotency marker POU5F1 was downregulated (Supplementary Fig. 1g,h). Therefore, siPOU5F1 was included in all subsequent experiments, unless otherwise noted.

### Identification of TFs that induce pretubular aggregate-like cells

The mature nephron consists of not only CDH16^+^ tubular cells, but also CDH16^−^ epithelial cells which include PODXL^+^ (Podocalyxin) podocytes – an important cell type responsible for blood filtration (Supplementary Fig. 2a). The overexpression of HNF1A alone differentiated hESCs into CDH16^+^ cells, but not PODXL^+^ or NPHS1^+^ podocyte cells (Supplementary Fig. 2b)^30^. This prompted us to identify TFs that could induce hESCs into pretubular aggregates, which are the precursors of both CDH16^+^ tubular cells and PODXL^+^ podocytes during kidney development^31,32^. To infer which candidate TFs to use, we repeated the *in silico* analyses described above and identified 17 TF candidates that upregulated the pretubular markers PAX2, PAX8, LHX1, WT1, and HNF1B (Supplementary Fig. 2c)^31,33–36^.

Among the 17 TFs, we first tested GATA3, which upregulated all the aforementioned markers except PAX8 *in silico* (Supplementary Fig. 2d). The combination of syn-HNF1A and syn-GATA3 enhanced the expression of pretubular aggregate markers and CDH16 (Supplementary Fig. 2e). Addition of syn-GATA1 further enhanced the effects of syn-HNF1A and syn-GATA3 on LHX1 expression (Supplementary Fig. 2f), suggesting that HNF1A, GATA3 and GATA1 can be used to differentiate hESCs into PAX8^+^LHX1^+^ pretubular aggregate-like cells. Furthermore, immunohistochemical analyses confirmed the induction of LHX1 and PAX8 expression by syn-HNF1A and syn-GATA3, which was further enhanced by the addition of syn-GATA1 (Supplementary Fig. 2g). Additionally, qRT-PCR analysis showed that co-transfection of syn-HNF1A, syn-GATA3, and syn-GATA1 increased the expression of BRN1 and CDH16 - distal tubular markers on day 2 of differentiation^37^; on the other hand, the expression of NPHS1, the podocyte marker, was not induced (Supplementary Fig. 2h).

### A cocktail of four TFs efficiently induces SIX2-positive nephron progenitor cells

**Figure 2:**
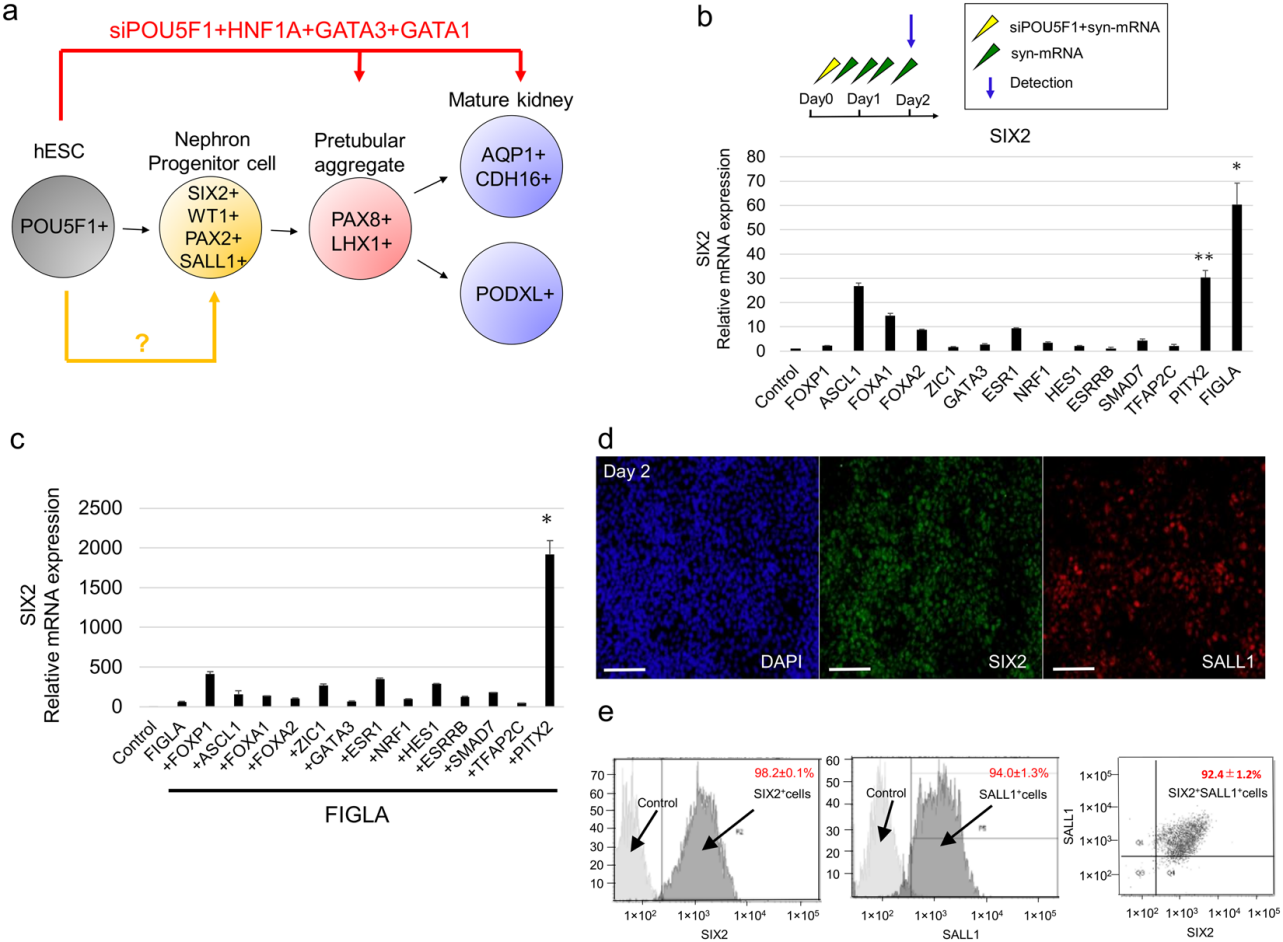
A cocktail of four TFs efficiently induces SIX2 positive nephron progenitor cells. (**a**) Diagram of differentiation into nephron progenitor cell. (**b**) Relative mRNA expression levels of SIX2 after transfecting candidate TF-encoding mRNAs into hESCs. Data represent mean + s.e.m. (**c**) Relative mRNA expression levels of SIX2 after transfecting FIGLA and other candidate TF-encoding mRNAs into hESCs. Data represent mean + s.e.m. (**d**) Immunocytochemistry for SIX2, SALL1, markers of nephron progenitor cells, on day 2 in cells differentiated from hPSCs using protocol depicted in. Scale bar, 100 μm. (**e**) Flow cytometry for SIX2, SALL1 in hESCs on day 2. Samples stained with secondary antibodies alone were used as controls. P-values were determined by a Student’s t-test. *P < 0.05; **P < 0.01.

The lack of podocyte marker expression further prompted us to explore the possibility of inducing the precursor of pretubular aggregates, that is, multipotent nephron progenitor cells (NPCs) which express SIX2, WT1, PAX2, and SALL1 (Fig. 2a)^38^. We repeated our TF candidate selection process *in silico* and identified 14 TF candidates which upregulate SIX2, WT1, PAX2, and SALL1 (Supplementary Fig. 3a). Then, we tested each of the synthetic mRNAs encoding for the 14TFs and found that syn-FIGLA strongly upregulated SIX2 expression (Fig. 2b).

We then transfected hESCs with syn-FIGLA and a cocktail of 3 pretubular-aggregate-inducing TFs (syn-HNF1A, syn-GATA3 and syn-GATA1), and found that the addition of syn-FIGLA decreased the expression of pretubular and tubular markers - PAX8, LHX1 and CDH16 (Supplementary Fig. 3b). These findings indicate that, although syn-FIGLA enhances SIX2 expression, its combination with pretubular aggregate-inducing TFs (syn-HNF1A, syn-GATA3, and syn-GATA1) is not beneficial presumably because syn-FIGLA sustains the stemness of NPCs. We, therefore, strategized to develop a two-step protocol to induce nephron epithelial cells through NPC induction.

To further increase NPC induction efficiency, we tested additional TFs and found that the addition of syn-PITX2 to syn-FIGLA strongly enhanced the expression of SIX2, whereas the addition of other TFs to those two TFs did not increase SIX2 expression (Fig. 2c, Supplementary Fig. 3c). Furthermore, the addition of syn-ASCL1 and syn-TFAP2C to the combination of syn-FIGLA and syn-PITX2 led to increased expression of other NPC markers such as PAX2 and WT1 (Supplementary Fig. 3d,e) as well as significant morphological changes of cells compared to control cells (Supplementary Fig. 3f). Moreover, immunocytochemical analysis revealed that these differentiated cells expressed SIX2 and SALL1 (Fig. 2d), and quantification by flow cytometry revealed that 92% of the differentiated cells were double-positive for SIX2 and SALL1 (Fig. 2e). To evaluate the capacity of these SIX2^+^ cells to form tubule-like branches *in vitro*, the cells were cultured for 24 hours on Matrigel directly placed on mitotically-inactivated Wnt4-producing cells (NIH3T3-Wnt4)^23,24,39^. Co-culture with the NIH3T3-Wnt4 cells substantially promoted branching formation, compared with co-culture with the NIH3T3 control cells that do not express Wnt4 (Supplementary Fig. 3g). Furthermore, immunostaining also showed that these structures partially expressed early renal vesicle markers - JAG1 and CDH6 (Supplementary Fig. 3h), suggesting that Wnt4 facilitated the mesenchymal to epithelial transition of the SIX2^+^SALL1^+^ cells.

### Sequential transfections of the TF cocktails along with syn-EMX2 is essential for epithelialization

**Figure 3:**
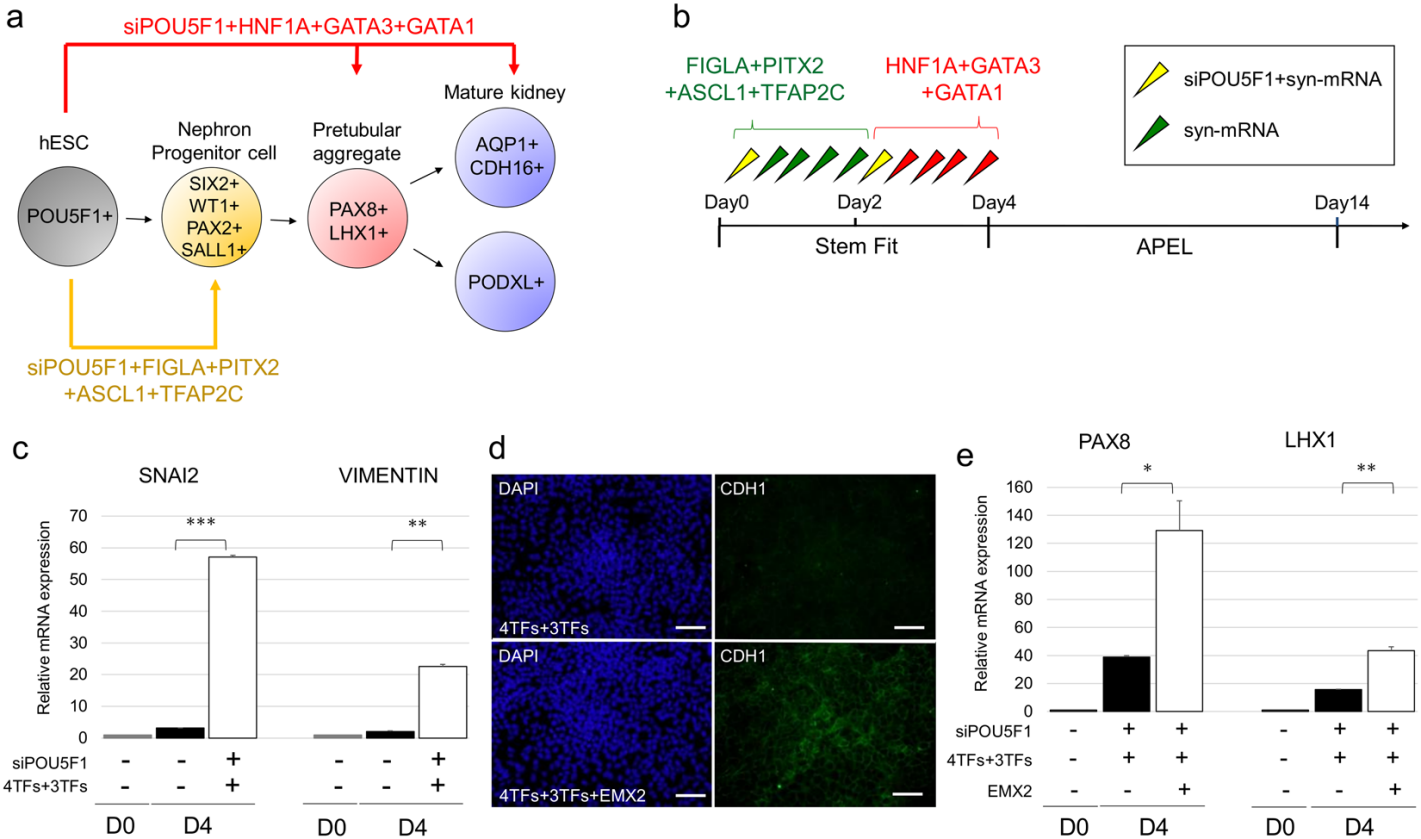
Sequential transfection of a cocktail of TFs along with EMX2, stimulating factor of mesenchymal-to-epithelial transition, is essential for epithelialization. (**a**) Diagram of differentiation. (**b**) Schematic of the tentative differentiation protocol from hESCs. (**c**) Relative mRNA expression levels of SNAI2 and VIMENTIN, mesenchymal markers, on day 4 in cells differentiated from hPSCs using protocol depicted in; 4TFs, syn-FIGLA, syn-PITX2, syn-ASCL1, and syn-TFAP2C; 3TFs, syn-HNF1A, syn-GATA3, and syn-GATA1. (**d**) Immunocytochemistry for CDH1, epithelial marker, on day 4 in cells differentiated from hPSCs after transfecting 4TFs + 3TFs and 4TFs + 3TFs + EMX2; 4TFs, syn-FIGLA, syn-PITX2, syn-ASCL1, and syn-TFAP2C; 3TFs, syn-HNF1A, syn-GATA3, and syn-GATA1. Scale bar, 100 μm. (**e**) Relative mRNA expression levels of PAX8 and LHX1, markers of pretubular aggregate, on day 4 in cells differentiated from hPSCs after transfecting 4TFs + 3TFs and 4TFs + 3TFs + EMX2; 4TFs, syn-FIGLA, syn-PITX2, syn-ASCL1, and syn-TFAP2C; 3TFs, syn-HNF1A, syn-GATA3, and syn-GATA1. Values shown are the means ± SEM. P-values were determined by a Student’s t-test. *P < 0.05; **P < 0.01; ***P < 0.001.

The cocktails of TFs presented thus far promote the differentiation of hESCs into pretubular aggregate-like cells and NPCs, but not kidney epithelial cells. Therefore, we modified the differentiation protocol to be sequential transfections with two TF cocktails: the transfection of hESCs with the first cocktail of TFs (syn-FIGLA, syn-PITX2, syn-ASCL1 and syn-TFAP2C) to induce SIX2^+^SALL1^+^ NPCs; the transfection of the NPCs with the second cocktail of TFs (syn-HNF1A, syn-GATA3 and syn-GATA1) to induce pretubular aggregate-like cells and nephron epithelial cells (Fig. 3a,b). This 2-step protocol led to the increased expression of the mesenchymal markers SNAI2 and VIMENTIN (VIM)^12,40,41^, but decreased the expression of E-cadherin (CDH1), an epithelial marker^31^, on day 4 of the differentiation procedure (Fig. 3c,d). These findings suggested that the differentiation from renal mesenchymal cells to epithelial cells requires a mesenchymal-to-epithelial transition (MET) step. When we measured the expression of EMX2 and CCND1 - TFs associated with epithelialization reported in previous studies^42^, we found little or no expression of EMX2 by immunocytochemical analysis (Supplementary Fig. 4). Indeed, the addition of syn-EMX2 to the second cocktail for pretubular aggregate formation (syn-HNF1A, syn-GATA3, and syn-GATA1) resulted in the increased expression of CDH1 (Fig. 3d). Moreover, syn-EMX2 inclusion led to the further enhancement of the expression of PAX8 and LHX1 - markers of pretubular aggregate-like cells on day 4 (Fig. 3e).

### Stepwise administration of TF cocktails induces kidney tissues in 2D culture

**Figure 4:**
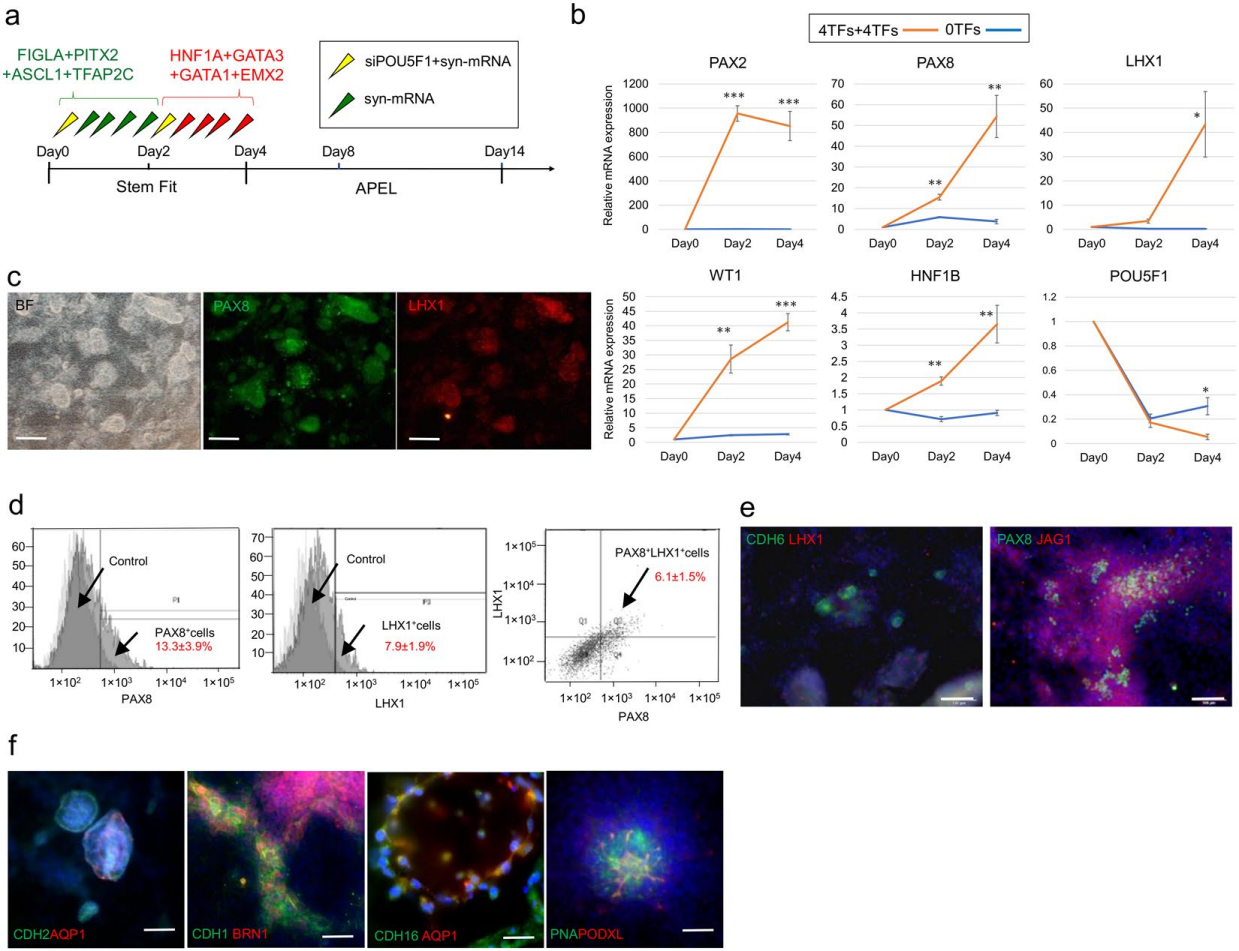
Stepwise TF cocktail administration induces nephrogenesis. (**a**) Schematic of the differentiation protocol from hESCs in 2D culture. (**b**) Relative mRNA expression levels of PAX2, PAX8, LHX1, WT1, HNF1B and POU5F1 differentiated from hPSCs. (**c**) Immunocytochemistry for PAX8, LHX1, markers of pretubular aggregate, on day 4 in cells differentiated from hPSCs using protocol depicted in. Scale bar, 200 μm. (**d**) Flow cytometry for PAX8, LHX1 in hESCs on day 4. Samples stained with secondary antibodies alone were used as controls. (**e**) Immunocytochemistry for CDH6, LHX1, PAX8 and JAG1, markers of renal vesicle, on day 8 in cells differentiated from hPSCs using protocol depicted in. Scale bar, 100 μm. (**f**) Immunocytochemistry for proximal tubule (CDH2, AQP1, CDH16), distal tubule (CDH1, BRN1), and podocyte (PODXL, PNA) on day 14. Scale bar, 50 μm. Values shown are the means ± SEM. P-values were determined by a Student’s t-test. *P < 0.05; **P < 0.01; ***P < 0.001.

Because the protein expression from syn-mRNAs is transient and peaks at around 8–12 hours before rapidly declining^18,43^, we optimized the transfection conditions and found that multiple transfections of each cocktail at an interval of 8 hours is sufficient to induce renal differentiation of hESCs. Subsequently, the cells were cultured in APEL medium for up to 14 days (Fig. 4a). Gene expression analysis revealed the increased expression of the pretubular aggregate markers PAX2, PAX8, LHX1, WT1, and HNF1B, suggesting pretubular aggregate induction, whereas the expression of the pluripotency marker POU5F1, was downregulated (Fig. 4b). Immunohistochemical analysis showed the expression of PAX8 and LHX1 by Day 4 (Fig. 4c). Quantification by flow cytometry revealed that approximately 6% of differentiated cells were double-positive for PAX8 and LHX1 (Fig. 4d). The low frequency of PAX8^+^LHX1^+^ cells is most likely due to the low transfection efficiency of the second TF cocktail. By Day 8, the differentiated cells expressed renal vesicle markers - CDH6, PAX8, and JAG1 (Fig. 4e)^31^. By day 14, renal vesicles spontaneously formed epithelial nephron-like structures. These structures expressed segmental markers of nephron-like structures, including proximal tubules (AQP1^+^ CDH16^+^), distal tubules (CDH1^+^ BRN1^+^), and glomerular podocytes (PNA^+^ PODXL^+^) (Fig. 4f). Subsequently, the differentiated cells started to detach from the culture dish, and could not be maintained afterwards.

### Generation of kidney-like structures in three-dimensional culture

**Figure 5:**
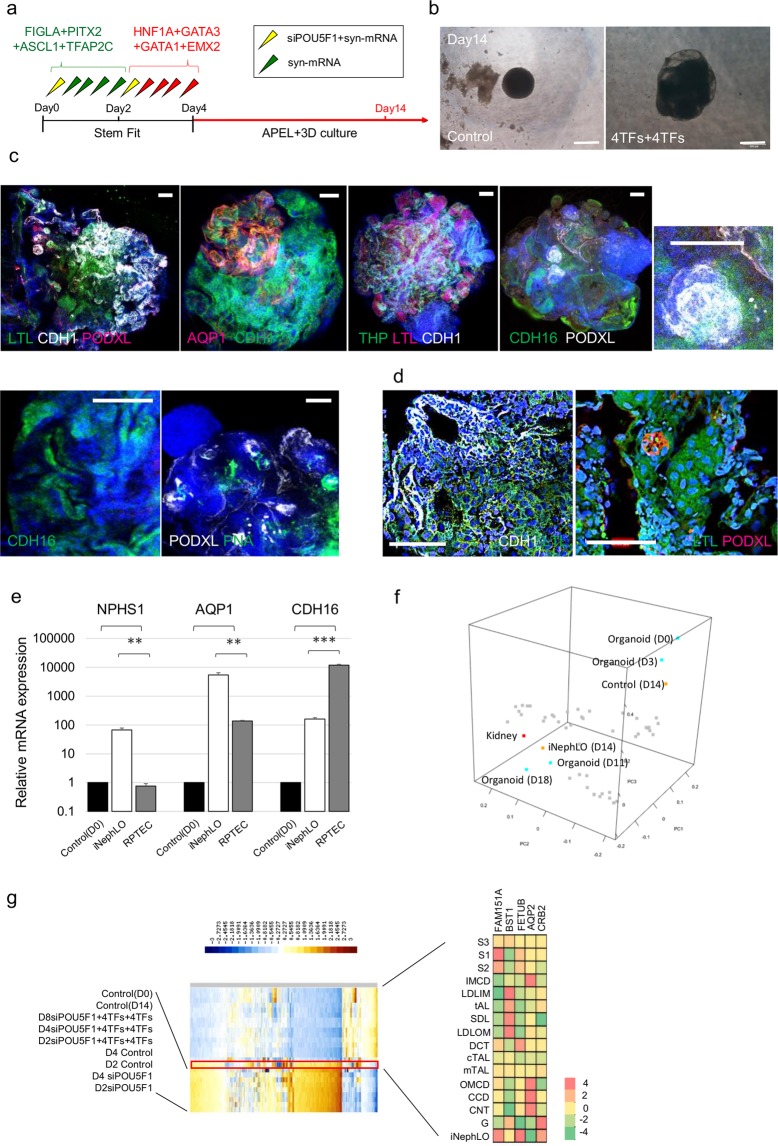
Generation of kidney tissues in 3D culture. (**a**) Schematic of the differentiation protocol from hESCs in 3D culture. (**b**) Bright-field imaging of the tissues that formed in culture after cells were resuspended on day4, transferred to ultra-low-attachment 96-well plates and studied on day14; Control, corresponding control cell tissues, 4TFs + 4TFs, syn-FIGLA, syn-PITX2, syn-ASCL1, syn-TFAP2C, syn-HNF1A, syn-GATA3, syn-GATA1, and syn-EMX2. Scale bar, 500 μm. (**c**,**d**) Immunocytochemistry for proximal tubule (LTL, AQP1), distal tubule (CDH1, THP), and podocyte (PODXL, PNA) on day 14. (**c**) Low magnification. Scale bar, 100 μm. (**d**) High magnification Scale bar, 50 μm. (**e**) Relative mRNA expression levels of segment-specific marker, compared with iNephLOs and RPTECs; Control, Day0 of undifferentiated hESCs. Data represent mean + s.e.m. (**f**) Principal component analysis of transcriptome data from iNephLOs (D14), corresponding Control Tissues (D14), kidney tissues (D0, D3, D11, and D18) reported previously (GSE70101), and other human organs (shown as grey dots: Testis, Lung, Heart, Liver, Tongue, Skeletal Muscle, Spinal cord, Whole brain, Hypothalamus, Brain thalamus, Brain amygdala, Prefrontal cortex, Occipital lobe, Pons, Cingulate cortex, Medulla oblongata, Parietal lobe, Temporal lobe, Cerebellum, Smooth muscle, Tonsil, Lymph node, Thymus, Prostate, Thyroid, Uterus, Salivary gland, Pituitary, Pancreas, Adrenal cortex, Skin, Appendix, Ovary, Myeloid, Monocytes, Whole blood, Dendritic cells, B cells, T cells, B lymphoblasts, Endothelial cells, Bone marrow, and Early erythroid). (**g**) Heatmap of RNA-seq expression analysis in rat kidney segments, control cell tissues and iNepLOs (left); S1–3, proximal tubule, first to third segment; IMCD, inner medullary collecting duct; LDLIM, long descending limb, inner medulla; tAL, thin ascending limb; SDL, short descending limb of the loop of Henle; LDLOM, long descending limb, outer medulla; DCT, distal convoluted tubule; cTAL, cortical thick ascending limb; mTAL, medullary thick ascending limb; OMCD, outer medullary collecting duct; CCD, cortical collecting duct; CNT, connecting tubule. A table illustrating the expression patterns along the different nephron segments (right); FAM151A (specific markers for proximal tubule); BST1 (specific markers for loop of Henle); FETUB (specific markers for distal tubule); AQP2 (specific markers for collecting duct); CRB2 (specific markers for glomeruli). Values shown are the means ± SEM. P-values were determined by a Student’s t-test. **P < 0.01; ***P < 0.001.

The difficulty of maintaining cell culture after day 14 prompted us to explore using three-dimensional (3D) cell culture, which has been successfully used to enhance the differentiation of cells to the renal lineage^44^. After completing the sequential transfections of two cocktails of TFs by day 4, we dissociated and re-plated the cells (corresponding to the pretubular aggregate stage), and maintained them in 3D-suspension culture for up to 14 days (Fig. 5a). The tissues grew rapidly and were much larger than corresponding control cell tissues by day 14 (Fig. 5b). Whole-mount immunostaining of tissues at day 14 showed tubule-like structures containing nephron features with the tissues similar to glomerulus and tubule. Expression of proximal tubule markers (LTL, AQP1, CDH16), distal tubular markers (CDH1, THP, CDH16), and podocyte markers (PNA, PODXL) were observed (Fig. 5c,d, Supplementary Fig. 5a, Supplementary video 1). These findings demonstrate that the maintenance of the pretubular aggregates in 3D-suspension culture resulted in kidney-like tissue structures, named induced nephron-like organoids (iNephLOs).

To compare iNephLOs to RPTECs, we measured expression levels of NPHS1, AQP1, and CDH16 by qRT-PCR. Both iNephLOs and RPTECs showed higher expression of AQP1 and CDH16 than the control undifferentiated hESCs, whereas NPHS1 was expressed only in iNephLOs (Fig. 5e), indicating that iNephLOs contain podocytes and proximal tubular cells. Considering the fact that iNephLOs consist of multiple cell types including podocytes and proximal tubular cells, the higher expression of AQP1 in iNephLOs than in RPTECs suggests that proximal tubular cells in iNephLOs have more functional phenotypes than primary culture of RPTECs. To further characterize iNephLOs, we also carried out global gene expression (transcriptome) profiling by RNA sequencing. The principal component analysis of transcriptome data showed that both iNephLO and kidney organoids generated using growth factor induction^10^ were more similar to adult kidney than any other human organs (Fig. 5f, Supplementary Data 1). The direct comparison between iNephLOs to kidney organoids generated using growth-factor induction also demonstrated that the expression of some of the segment-specific markers normalized by internal control resembled each other (Supplementary Fig. 5b,c). Furthermore, when compared to rat kidney segment transcriptome data, the transcriptome of iNephLOs showed similarity to proximal tubules (S1, S2, S3), distal tubules, and glomerulus (Fig. 5g).

### Functional assays of iNephLOs

**Figure 6:**
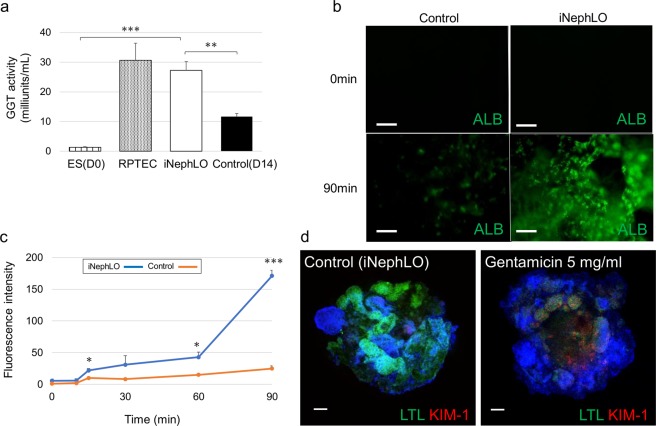
Function assays for iNephLOs. (**a**) The concentration of p-nitroaniline (pNA) produced by hESCs (Day0), RPTECs, iNephLOs, and corresponding control cell organoids (shown as Control) were measured and normalized to the cell number. (n = 3). (**b**) Representative images of corresponding control cell organoids (shown as Control) and iNephLOs incubated with 488-conjugated albumin for the indicated time periods. Scale bar, 50 μm. (**c**) Quantification of albumin uptake in iNephLOs and control over a 90 min time course, n = 3 visual fields per time point. (**d**) Representative immunohistochemistry of iNephLOs treated with Gentamicin 5 mg/ml from day14 to 16. Scale bar, 100 μm. Values shown are the means ± SEM. P-values were determined by a Student’s t-test. *P < 0.05; **P < 0.01; ***P < 0.001.

To demonstrate the functionality of iNephLOs, we analyzed the activity of γ-glutamyl transferase (GGT), which is known to be expressed in the proximal tubule^45,46^ and reflects the functional characteristic of proximal tubular cells. The release of p-nitroanilline, which is the product of GGT activity, by the iNephLOs was as high as in the RPTECs (Fig. 6a). Next, we examined the endocytotic uptake of proteins, including albumin, a crucial function for proximal tubular cells^29^. iNephLOs showed the more efficient uptake of albumin conjugated with a green fluorescent compared to the control, indicating their endocytic functionality (Fig. 6b,c). Furthermore, to demonstrate that iNephLOs can be used to study kidney injury and drug nephrotoxicity, we treated iNephLOs with gentamicin (5 mg/ml), a commonly used antibiotic agent, on day 14 of the differentiation for 48 hours. Kidney injury molecule-1 (KIM-1), a specific biomarker for proximal tubular injury^47^, markedly increased in LTL^+^ cells in iNephLOs treated with gentamicin (Fig. 6d). These findings demonstrate that iNephLOs possess some level of functionality of kidneys.

## Discussion

In this study, we have described a new method of differentiating hPSCs to nephron-like structures via NPC induction by stepwise transfection of syn-mRNAs encoding TFs. The five consecutive transfections of four TFs (syn-FIGLA, syn-PITX2, syn-ASCL1, and syn-TFAP2C) convert hPSCs into NPCs more rapidly and efficiently than the latest protocols^7,11^, and subsequently, the five consecutive transfections of four TFs (syn-HNF1A, syn-GATA3, syn-GATA1, and syn-EMX2) further convert them into nephron-like structures.

Some of the TFs identified in this study are also implicated in kidney development in previous developmental studies. For example, all four TFs (FIGLA, PITX2, ASCL1, and TFAP2C) in the first TF cocktail are expressed in the mesonephros, an early precursor of the kidney during development^48–51^, although the expression of these TFs in the metanephros has not been reported. GATA3 and EMX2 in the second TF cocktail are expressed in the ureteric bud, and HNF1A is a well-known factor expressed in S-shaped bodies - the early segmented nephron structures^52^. The role of GATA1 in the kidney is unknown, but some studies have reported that GATA1 regulates SIX2 expression by acting on the SIX2 promoter^53^. The absence of these TFs (HNF1A, GATA3, GATA1, and EMX2) is reported to affect kidney development in knock-out mouse models and human diseases^54–57^. Interestingly, FIGLA, the SIX2-inducing TF, downregulates CDH16 expression induced by HNF1A. This may be explained by the role of SIX2 in the suppression of nephrogenesis^58^. The effects of TFs revealed in this study may help to elucidate the roles of each TF in kidney development.

To the best of our knowledge, this is the first demonstration that hESCs can be converted into NPCs and nephron-like tissues by using only syn-mRNAs encoding TFs and without using any growth factors whose effects depend on their receptor expression. Furthermore, the combination of TFs with 3D-suspension culture results in iNephLOs in 14 days of differentiation, which contains nephron-like tissues with characteristics of glomeruli, proximal tubules, and distal tubules. RNA-seq analyses demonstrated that iNephLOs closely resemble human adult kidneys and kidney organoids previously reported^10^. However, some segment-specific markers such as the podocyte marker CRB2, proximal tubular marker HNF1B, distal tubular marker KCNJ1 showed lower expression levels in iNephLOs compared to the kidney organoids. Furthermore, immunohistochemical analyses could not show contiguous tubules clearly, suggesting that these tubules are not thoroughly organized. Therefore, iNephLOs seem to be less mature than the previously reported kidney organoids. One possible way to overcome this shortcoming may be to increase the transfection efficiency of the second set of TF cocktail (HNF1A, GATA3, GATA1 and EMX2), which currently produces PAX8^+^LHX1^+^ pretubular aggregates inefficiently (only 6% of cells compared to approximately 75% of cells by one protocol previously reported^7^). However, this may require new development or further optimization of RNA transfection methods, which can achieve highly efficient transfection into partially differentiated cells. Alternatively, it is conceivable to combine the current syn-TF-based method and previously reported growth factor- and/or small molecule-based methods. For example, after the transfection of the first set of TF cocktail (FIGLA, PITX2, ASCL1, TFAP2C), on day 2, differentiating cells (corresponding to the nephron progenitor cell stage) can be exposed to CHIR99021 and FGF9 for further differentiation as previously reported^7,10^. However, this may also require further optimization of cell culture conditions.

Our strategy uses syn-mRNAs encoding for TFs (syn-TFs) for the forced expression of TFs. As we have also shown in our earlier work^17–20,59^, syn-TF-based differentiation of hPSCs is footprint-free, because mRNAs are directly translated into proteins to function and are not converted into DNAs, which could cause unfavorable genome modification^60^. The forced expression of combinations of TFs directly manipulates the gene regulatory network, which is most likely the reason for the faster and more efficient differentiation of hPSCs into NPCs and kidney cells compared to growth factor- and small molecule-based methods. The method to generate iNephLOs presented in this study may help to achieve goals in clinical applications, including *in vitro* drug testing and screening, and cell/organ replacement therapy.

## Materials and Methods

### Cells and culture conditions

SEES3 human ES cells^61^ were kindly provided by Dr. Hidenori Akutsu from the National Center for Child Health and Development, Japan. SEES3 cells were cultured under feeder-free conditions in StemFit AK03 or AK03N medium (Ajinomoto) on Laminin511 (iMatrix-511: Nippi, #892012) coated dishes.

### Generation of synthetic messenger RNAs

The procedure for generation of synthetic mRNAs is described in previous reports^18^. The purified synthetic mRNA obtained was size verified and stored as aliquots at −80 °C until use. The accession numbers for TFs are listed in Supplementary Table 1.

### Differentiation of SEES3

SEES3 cells seeded on Laminin511 were washed with PBS (Nacalai tesque, #14249-24) and detached with mixture of 0.5 mM EDTA (Kanto Chemical, #14566-08) and TrypLE Select (1:1, Thermo Fisher Scientific, #A1285901). Cells were then seeded in 24-well tissue culture plates (Costar, #3526) coated with Laminin511 at a density of 8.0 × 10^4^ cells per well in StemFit AK03 or AK03N medium supplemented with the ROCK inhibitor Y27632 (10 μM) (Wako, #251-00514). After 40 hr, before mRNA transfection, the medium was replaced with StemFit AK03 or AK03N containing B18R (200 ng/ml final concentration) (Fisher scientific, #509332), a protein that binds to type 1 interferon to prevent toxicity during mRNA transfection and increase the viability of cells^62^. RNA transfections were performed with Lipofectamine Messenger Max (Life technologies, LMRNA003) according to manufacturer’s instructions. In brief, 1 μ g mRNA cocktail and 1 μl siPOU5F1 (20 nM final concentration) (Life technologies, #4392420) in OptiMEM (Life technologies, #31985070) was mixed with 2 μl Messenger Max in OptiMEM and incubated at RT for five minutes for complex formation. Complexes were then added dropwise to wells. Medium was replaced three hours after transfection with StemFit AK03 or AK03N containing B18R. We transfected each cocktail a total of five times with an interval of eight hours between the transfections. We transfected Cocktail 1(syn-FIGLA, syn-PITX2, syn-ASCL1, and syn-TFAP2C), followed by Cocktail 2(syn-HNF1A, syn-GATA3, syn-GATA1, and syn-EMX2). The first transfection of each cocktail had siPOU5F1 along with mRNA. After a three-hour incubation period after the final transfection, the medium was replaced with APEL basal medium (STEMCELL Technologies, #ST-05210). The medium was then replaced every 2 days.

### Differentiation of transgenic hESC lines

The procedure for establishment of transgenic hESC line was described in previous reports^63^. HNF1A-inducible hESCs were cultured in StemFit AK03 or AK03N medium with 1 mg/ml of doxycycline (Sigma, #D9891-1G) and 1 mg/ml puromycin (InvivoGen, #ant-pr-1) for 2 days. The medium was then changed to REGM supplemented with REGM SingleQuots (Lonza, CC-4127) containing 0.5% fetal bovine serum (FBS), and 0.1% recombinant human epidermal growth factor, insulin, hydrocortisone, epinephrine, triiodothyronine, and transferrin by volume. Cells were then incubated for an additional 3 days without doxycycline.

### Kidney tissue formation

On day 4 of differentiation, hESCs after final transfection (corresponding to the pretubular aggregate stage) were dissociated with a mixture of 0.5 mM EDTA and TrypLE Select, and replated on 96-well, round bottom, ultra-low-attachment plates (Corning, #7007) at 5.0 × 10^4^ cells per well in the basic APEL medium supplemented with Y27632. Cells were spun down at 500 g for 30 s, and incubated for 2 days. Subsequently, the medium was changed to the basic APEL medium without additional factors. Half of the culture medium volume was refreshed with new medium every 2 days for 10 days (a total of 14 days).

### Production of antibodies

The procedure for production of antibodies is described in previous reports^64^. Polyclonal antibodies were against a partial length (a.a. 544-744) of human CDH16. The purified protein was used to immunize rabbits.

### Western blot analysis

The cultured HNF1A-inducible cell line was dissociated and similar numbers of cells were pelleted. The cell pellets were washed twice with PBS, and lysed into RIPA buffer (Sigma, #R0278) according to the manufacturer’s instructions. The proteins were mixed with Sample buffer (Wako, # 198-13282), denatured, separated by SDS-PAGE on Mini-PROTEAN TGX Gels (Bio-Rad, #456-1086), and were transferred to Immun-Blot PVDF membranes (Bio-Rad, #162-0177). The membranes were blocked for 1 h with 5% skim milk in Tris-buffered saline containing 0.1% Tween-20 (TBST), washed in TBST, and then incubated with the primary antibodies against HNF1A (Abcam, ab96777; 1:500) and β -ACTIN (Cell Signaling, #4970; 1:1000) in 2% BSA/TBST overnight at 4 °C. The membranes were washed three times in TBST and then incubated with horseradish peroxidase-conjugated anti-rabbit antibodies (GE Healthcare, NA934) (1:2000) for 1 h at RT. The immunoreactivity was detected using an ECL Prime Western Blotting Detection Kit (GE Healthcare, RPN2232) and LAS-4000 (Fujifilm).

### Immunocytochemistry

Cells were rinsed with PBS and fixed with 4% paraformaldehyde for 10 min at RT. Fixed cells were washed three times in PBS and blocked with 0.3% Triton X-100 and 5% normal bovine serum for 30 min at RT. The cells were then incubated with the primary antibodies overnight at 4 °C in 0.3% Triton X-100 and 5% normal bovine serum. After washing three times in PBS, the cells were incubated with Alexa Fluor 488- or 594-conjugated secondary antibodies (1:1,000) (Life Technologies) in 0.3% Triton X-100 and 5% normal bovine serum for 1 hr at RT. DAPI (Sigma, #D8417) was added to stain the nucleus. The primary antibodies used for immunocytochemistry are listed in Supplementary Table 2. Immunofluorescence was photographed using Olympus microscope (IX73) or Delta Vision Elite microscope (GE Healthcare).

### Whole-mount immunohistochemistry of 3D tissues

Tissues were rinsed with PBS and fixed with 4% paraformaldehyde overnight at RT in a 96-well plate. Fixed tissues were washed three times in 0.1% Triton X-100 and blocked with 1% Triton X-100, 5% normal bovine serum, 0.2% skim milk and 10% DMSO twice for 1 hr at RT. After washing three times in 0.1% Triton X-100, the tissues were incubated with the primary antibodies in 1% Triton X-100, 5% normal bovine serum, 0.2% skim milk and 5% DMSO over two nights at 4 °C on a shaker. After washing off the unbound primary antibodies with 0.1% Triton X-100 three times for 30 min each, with the third washing performed overnight at 4 °C. The tissues were incubated with Alexa Fluor 488-, 594-, or 647-conjugated secondary antibodies in 1% Triton X-100, 5% normal bovine serum and 0.2% skim milk overnight at 4 °C on a shaker, then washed with 0.1% Triton X-100 three times for 30 min each, with the third washing performed overnight at 4 °C. For immunostaining with biotinylated LTL (Vector Labs, #B-1325), Streptavidin/Biotin Blocking Kit (Vector Labs, #SP-2002) and Alexa Fluor 488-conjugated streptavidin (Life Technologies) were used according to manufacturer’s instructions. DAPI was added to stain the nucleus for more than 30 min. Subsequently, the tissues were fixed with 4% paraformaldehyde for 30 min at 4 °C, dehydrated with 50% ethyl alcohol for 1 hr, with 70% ethyl alcohol for 1 hr, with 100% ethyl alcohol for 1 hr twice at 4 °C, and cleared with ethyl cinnamate (Sigma, #112372-100 G) for 30 min at RT. Finally, the tissues were mounted with Vectashield (Vector Labs, #H-1200) and examined by confocal microscopy (Leica TCS SP8) and by using Imaris 3D software (Bitplane).

### Quantitative RT-PCR

Total RNA was isolated using a miRNeasy Mini Kit (Qiagen, #217004) and reverse-transcribed with the High-Capacity Reverse Transcription Kit (Applied Biosystems, #4368814) according to the manufacturer’s instructions. Real-time PCR was performed using a QuantiFast SYBR Green PCR Kit (Qiagen) according to the manufacturer’s protocol. The expression of mRNA was assessed by evaluating threshold cycle(CT) values. The CT values and relative expression levels were normalized by actin beta(ACTB), and the relative amount of mRNA specific to each of the target genes was calculated (n = 2–3 in each experiment). The primer sequences used for qRT-PCR are listed in Supplementary Table 3.

### Flow cytometry

Cells were dissociated with 0.5 mM EDTA/TrypLE Select for 10 min, and filtered through a 70-μm cell strainer (Corning, #352350). Cells were fixed with 4% paraformaldehyde for 15 min on ice and permeabilized with 0.3% Triton for 15 min on ice. Cells were then blocked with 5% donkey serum for 15 min and incubated with the primary antibodies (PAX8 1:2,500, LHX1 1:100, SIX2 1:1,000, SALL1 1:100) for 30 min. After washing three times with 0.1% BSA, cells were incubated with Alexa Fluor 488- or 594-conjugated donkey secondary antibodies (1:5,000) for 30 min on ice. Cells were then washed three times with 0.1% BSA. Flow cytometry was performed using BD FACSAria II (BD Biosciences).

### Directional RNA sequencing and data analysis

Total RNA was isolated from hESCs (Day 0), induced tissues (iNephLO, Day 14) and control organoids (Control, Day 14). Each 500 ng total RNA was used for library preparation using NEBNext Ultra Directional RNA Library Prep Kit for Illumina (New England BioLabs, #E7420S). The prepared library was quantified and subjected to 75-bp paired-end sequencing using MiSeq (Illumina). Frequency per kilo-base per million reads (FPKM) and frequency per million reads (FPM) was calculated using Tophat and Cufflinks software. Transcriptome data obtained from iNephLOs were compared with those obtained from kidney organoids reported previously (GSE70101, count data)^10^ and multiple human tissues (GSE1133, microarray intensity data)^65^. Transcriptome data obtained from iNephLOs were also compared with micro-dissected rat renal tubule segments (GSE56743, FPKM data)^66^. Principal component analysis (PCA) was performed by ExAtlas software (https://lgsun.irp.nia.nih.gov/exatlas/index.html)^67^ based on the single-factor analysis of variance (ANOVA). 3D plot was generated by R with rgl package (and R markdown and webGL were used for interactive 3D visualization). Control and marker gene expression levels were extracted from the normalized data, and were plotted as box- and dot-plots for iNephLO and Control, compared with the time-course data of control organoids (n = 3, respectively). For the heatmap with rat segmental data, each FPKM value wasused with quantile normalization.

### γ-Glutamyltransferase (GGT) activity

iNephLOs, primary non-neoplastic human renal proximal tubular epithelial cells (RPTEC; Lonza, #CC-2553), hESCs (Day0), and corresponding control cell organoids (shown as Control) were homogenized in 200 μL of ice-cold GGT Assay Buffer. γ-GGT activity was determined using γ-GGT activity colorimetric assay kit (Sigma, MAK089) according to the manufacturer’s instructions.

### Albumin uptake assay

iNephLOs and corresponding control cell organoids (shown as Control) were incubated with FITC-conjugated albumin (1 mg/ml, Life Technologies, A23015) at several timepoints. Subsequently, cells were washed five times with ice-cold PBS in order to stop endocytosis. Tissues were fixed with 4% paraformaldehyde (PFA) and photographed using Olympus fluorescence microscope (IX73).

### Cytotoxicity assay

iNephLOs were cultured in APEL basal medium supplemented with gentamicin 5 mg/ml (Sigma, #G1264) for 48 hr after day 14 of differentiation. Tissues were then fixed with 4% paraformaldehyde for 20 min for whole-mount immunohistochemistry.

### Statistical analysis

All data is presented as the means ± S.E.M. Group comparisons were analysed by the Student’s t-test. A P-value of < 0.05 was considered significant.

## Supplementary information

Supplementary Video 1

Supplementary Information

Supplementary Data 1
