# Supplementary material for: Induction of human pluripotent stem cells into kidney tissues by synthetic mRNAs encoding transcription factors
Source: Sci Rep. 2019 Jan 29;9:913. doi: 10.1038/s41598-018-37485-8 (PMC6351687; doi:10.1038/s41598-018-37485-8)
Supplement: Supplementary file 2 — Supplementary Information [file 41598_2018_37485_MOESM2_ESM.pdf]

Supplementary Information

Induction of human pluripotent stem cells into kidney tissues by synthetic mRNAs encoding transcription factors

Ken Hiratsuka[1,2], Toshiaki Monkawa[2, 3], Tomohiko Akiyama[1], Yuhki Nakatake[1], Mayumi Oda[1], Sravan Kumar Goparaju[1], Hiromi Kimura[1], Nana Chikazawa-Nohtomi[1], Saeko Sato[1], Keiichiro Ishiguro[1,4], Shintaro Yamaguchi[2], Sayuri Suzuki[2], Ryuji Morizane[2, 5, 6, 7], Shigeru B.H. Ko[1], Hiroshi Itoh[2], and Minoru S.H. Ko[1, *]

[1] Department of Systems Medicine, Keio University School of Medicine, 35 Shinanomachi, Shinjuku, Tokyo 160-8582, Japan
[2] Department of Nephrology, Endocrinology, and Metabolism, Keio University School of Medicine, 35 Shinanomachi, Shinjuku, Tokyo 160-8582, Japan
[3]Medical Education Center, Keio University School of Medicine, 35 Shinanomachi, Shinjuku, Tokyo 160-8582, Japan
[4] Institute of Molecular Embryology and Genetics, Kumamoto University, 2-2-1 Honjo, Chuo-ku, Kumamoto 860-0811, Japan
[5] Renal Division, Department of Medicine, Brigham and Women's Hospital, Boston, Massachusetts, USA
[6]Department of Medicine, Harvard Medical School, Boston, Massachusetts, USA
[7]Harvard Stem Cell Institute, Cambridge, Massachusetts, USA

*Correspondence to be addressed.

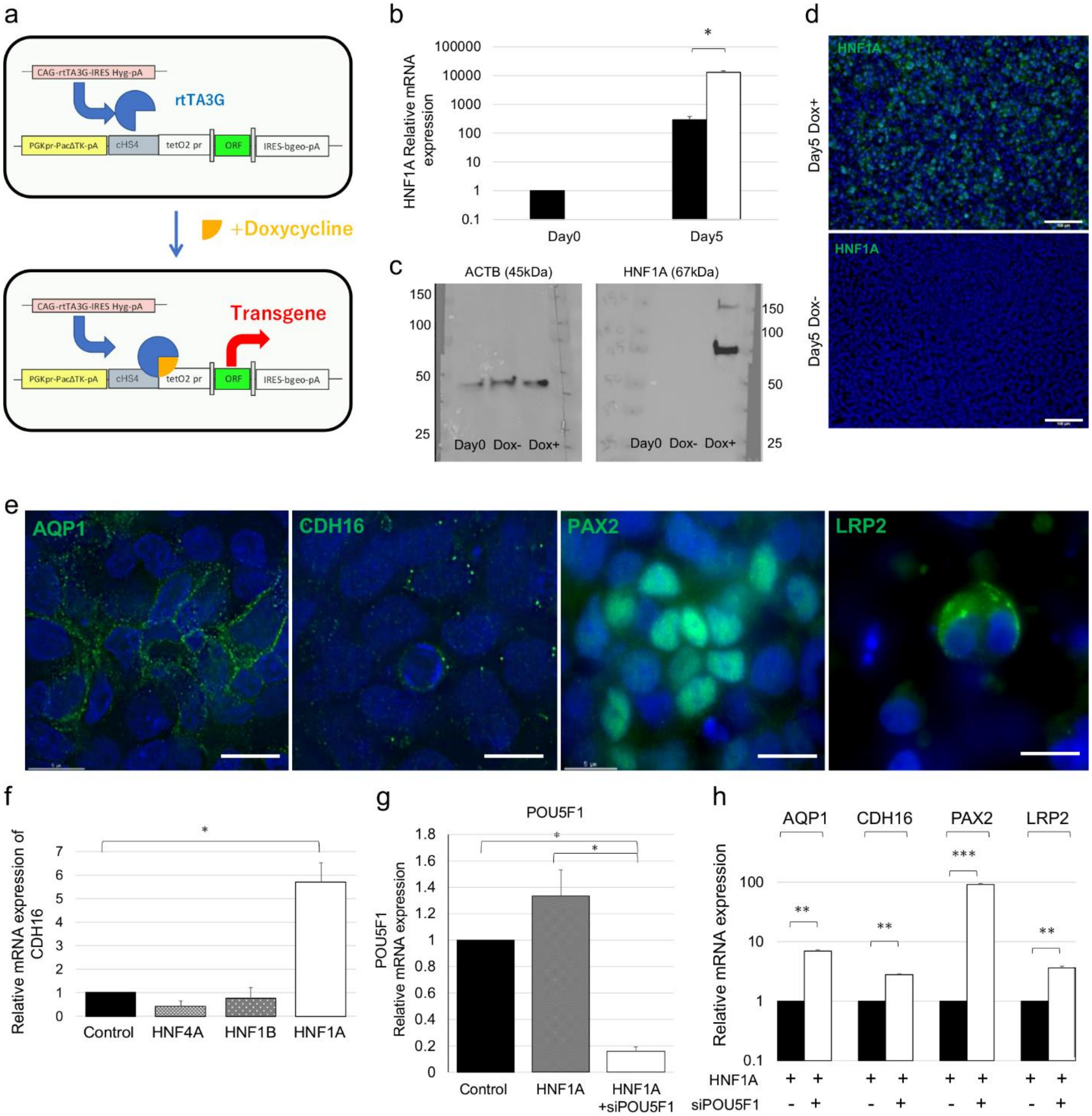

Supplementary Figure S1

(a) Establishment of tetracycline-inducible gene expression system in hESCs. (b) Relative expression levels of HNF1A was determined by real-time PCR for Day0 hESCs (black bar), Day5 transgene un-induced hESCs (black bar), and Day5 transgene HNF1A-induced hESCs (white bar). (c) Western blot analysis of HNF1A expression in Day0 hESCs (left), Day5 transgene un-induced hESCs (middle), and Day5 transgene HNF1A-induced hESCs (right). (d) Expression of HNF1A at Day5 after the activation of single transgene, HNF1A. (e) Expression of marker genes at Day5 after the activation of single transgene, HNF1A. (f) The human kidney master regulatory TF is different from that of other species. n = 2. Data represents mean +s.e.m. (g) The expression of POU5F1 was downregulated with siPOU5F1 treatment. n = 2. Data represent mean +s.e.m. (h) siPOU5F1 accelerates the differentiation of hESCs towards the renal lineage. Values shown are the mean ± SEM. P-values were determined by a Student's t-test. *$P < 0.05$; **$P < 0.01$; ***$P < 0.001$.

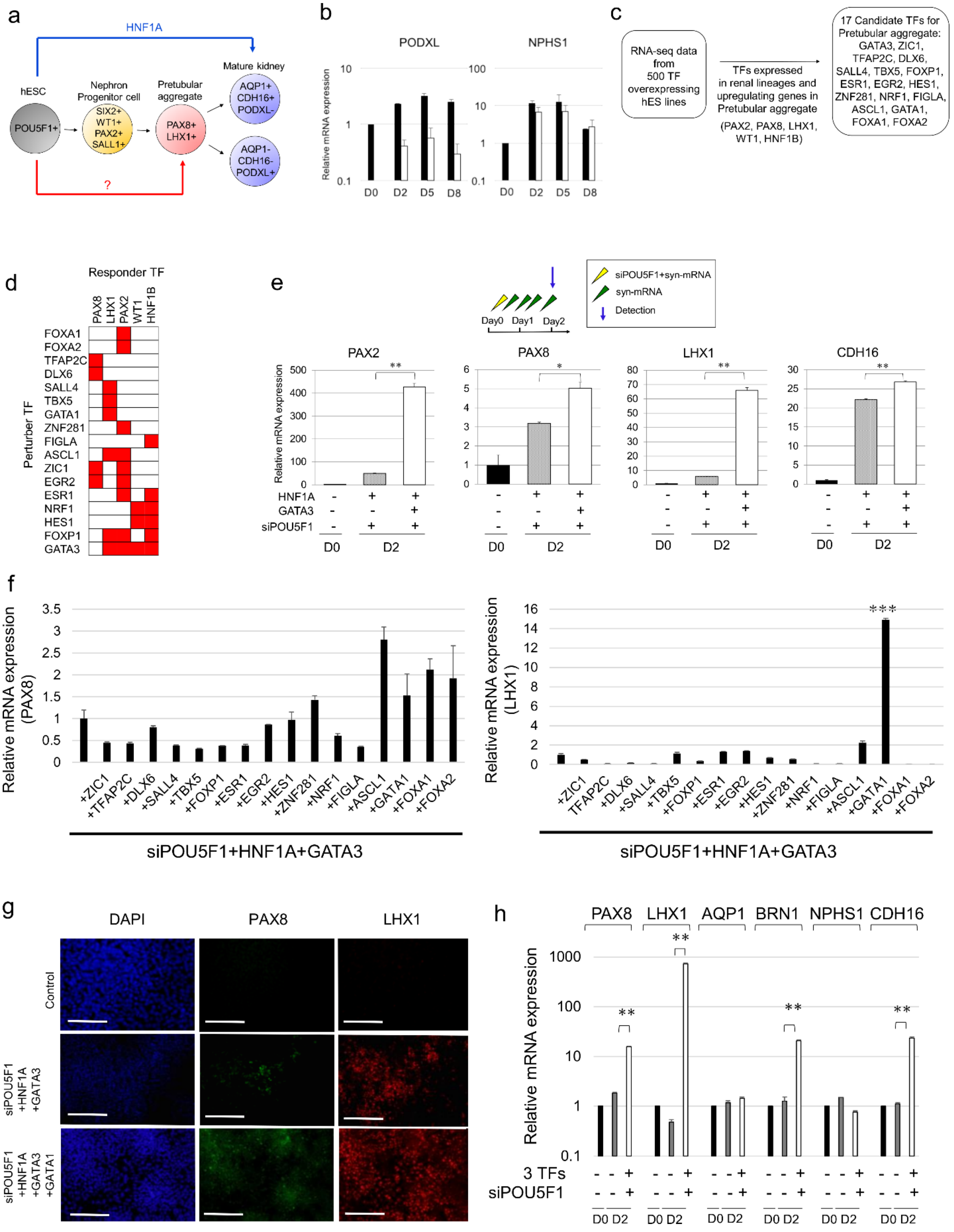
a
HNF1A
hESC
POU5F1+
Nephron Progenitor cell
SIX2+ WT1+ PAX2+ SALL1+
Pretubular aggregate
PAX8+ LHX1+
Mature kidney
AQP1+ CDH16+ PODXL-
AQP1- CDH16- PODXL+
?
b
PODXL
NPHS1
Relative mRNA expression
D0 D2 D5 D8
c
RNA-seq data from 500 TF overexpressing hES lines
TFs expressed in renal lineages and upregulating genes in Pretubular aggregate
(PAX2, PAX8, LHX1, WT1, HNF1B)
17 Candidate TFs for Pretubular aggregate: GATA3, ZIC1, TFAP2C, DLX6, SALL4, TBX5, FOXP1, ESR1, EGR2, HES1, ZNF281, NRF1, FIGLA, ASCL1, GATA1, FOXA1, FOXA2
d
Responder TF
PAX8 LHX1 PAX2 WT1 HNF1B
Perturber TF
FOXA1 FOXA2 TFAP2C DLX6 SALL4 TBX5 GATA1 ZNF281 FIGLA ASCL1 ZIC1 EGR2 ESR1 NRF1 HES1 FOXP1 GATA3
e
siPOU5F1+syn-mRNA
syn-mRNA
Detection
Day0 Day1 Day2
PAX2
PAX8
LHX1
CDH16
Relative mRNA expression
HNF1A
GATA3
siPOU5F1
D0 D2
f
Relative mRNA expression (PAX8)
Relative mRNA expression (LHX1)
+ZIC1 +TFAP2C +DLX6 +SALL4 +TBX5 +FOXP1 +ESR1 +EGR2 +HES1 +ZNF281 +NRF1 +FIGLA +ASCL1 +GATA1 +FOXA1 +FOXA2
siPOU5F1+HNF1A+GATA3
siPOU5F1+HNF1A+GATA3
g
DAPI
PAX8
LHX1
Control
siPOU5F1 +HNF1A +GATA3
siPOU5F1 +HNF1A +GATA3 +GATA1
h
PAX8 LHX1 AQP1 BRN1 NPHS1 CDH16
Relative mRNA expression
3 TFs
siPOU5F1
D0 D2

Supplementary Figure S2

(a) Diagram of differentiation into pretubular aggregate. (b) Relative mRNA expression levels of podocyte markers (PODXL, NPHS1) in transgene HNF1A-induced hESCs (white bars) and in transgene un-induced hESCs (black bars). Data represent mean +s.e.m. (c) Flowchart of an approach to identify pretubular aggregate-specific TFs. (d) 17 candidate TFs that upregulate pretubular aggregate markers. (e) Relative mRNA expression levels of pretubular aggregate markers (PAX2, PAX8, LHX1), and CDH16 after transfecting HNF1A and GATA3 mRNAs into hESCs. Data represent mean +s.e.m. (f) Relative mRNA expression levels of pretubular aggregate markers (PAX8, LHX1). The synergistic effect was observed after transfecting GATA1 in addition to HNF1A and GATA3. Expression of PAX8 and LHX1 are shown as fold induction relative to the expression levels after transfecting HNF1A and GATA3. Data represents mean +s.e.m. (g) Immunocytochemistry for PAX8 and LHX1 on day 2 of differentiation. Control indicates corresponding control cells without any TFs. Scale bar, 200 µm. (h) Relative mRNA expression levels of pretubular aggregate markers (PAX8, LHX1), AQP1, BRN1, NPHS1, and CDH16 after transfecting HNF1A, GATA3 and GATA1 mRNAs into hESCs. Podocyte marker, NPHS1 is not upregulated after transfecting 3TFs for 2 days. Data represent mean +s.e.m.

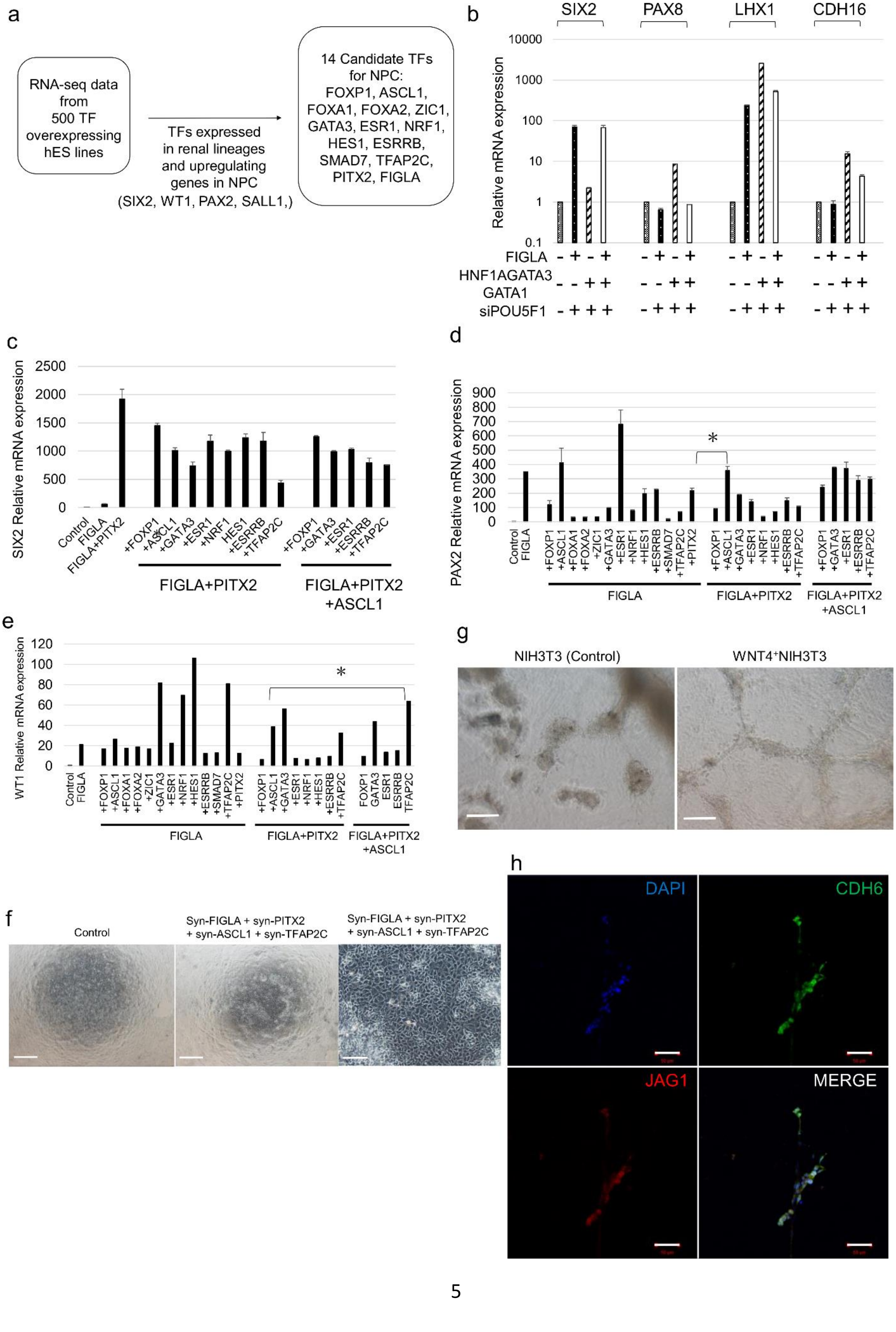
a
RNA-seq data from 500 TF overexpressing hES lines
TFs expressed in renal lineages and upregulating genes in NPC (SIX2, WT1, PAX2, SALL1,)
14 Candidate TFs for NPC: FOXP1, ASCL1, FOXA1, FOXA2, ZIC1, GATA3, ESR1, NRF1, HES1, ESRRB, SMAD7, TFAP2C, PITX2, FIGLA
b
SIX2
PAX8
LHX1
CDH16
Relative mRNA expression
FIGLA
HNF1AGATA3
GATA1
siPOU5F1
c
SIX2 Relative mRNA expression
Control
FIGLA
FIGLA+PITX2
FIGLA+PITX2
FIGLA+PITX2 +ASCL1
d
PAX2 Relative mRNA expression
FIGLA
FIGLA+PITX2
FIGLA+PITX2 +ASCL1
e
WT1 Relative mRNA expression
FIGLA
FIGLA+PITX2
FIGLA+PITX2 +ASCL1
g
NIH3T3 (Control)
WNT4+NIH3T3
f
Control
Syn-FIGLA + syn-PITX2 + syn-ASCL1 + syn-TFAP2C
Syn-FIGLA + syn-PITX2 + syn-ASCL1 + syn-TFAP2C
h
DAPI
CDH6
JAG1
MERGE

Supplementary Figure S3

(a) Flowchart of an approach to identify NPC specific TFs. (b) Relative mRNA expression levels of SIX2, PAX8, LHX1 and CDH16 after transfecting FIGLA in addition to 3TFs. PAX8, LHX1, CDH16 were down regulated in combination with FIGLA. Data represents mean +s.e.m. (c) Relative mRNA expression levels of SIX2 after transfecting FIGLA, PITX2 and other candidate TF-encoding mRNAs into hESCs. Data represent mean +s.e.m. (d) Relative mRNA expression levels of PAX2 after transfecting FIGLA and other candidate TF-encoding mRNAs into hESCs. Data represents mean +s.e.m. (e) Relative mRNA expression levels of WT1 after transfecting FIGLA and other candidate TF-encoding mRNAs into hESCs. Data represents mean +s.e.m. (f) Representative bright-field images on day 2 of differentiation. Control indicates corresponding control cells without any TFs. Morphological changes of induced NPCs compared to control cells. Scale bar, 500μm (Left, Middle) 100 μm (Right). (g) SIX2+SALL1+ cells formed branching structure in matrigel. Scale bar, 200μm. (h) Immunostaining showed branching structures partially expressed JAG1 and CDH6. Scale bar, 50μm. P-values were determined by a Student's t-test. *P < 0.05; **P < 0.01; ***P < 0.001.

a

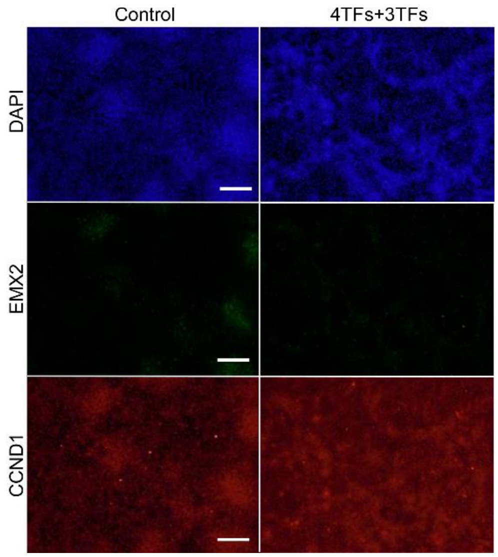

Supplementary Figure S4

Immunocytochemistry for EMX2 and CCND1, essential TFs for MET, on day 4 in cells differentiated from hPSCs using protocol depicted in; 4TFs, syn-FIGLA, syn-PITX2, syn-ASCL1, and syn-TFAP2C; 3TFs, syn-HNF1A, syn-GATA3, and syn-GATA1. Scale bar, 200 µm.

a

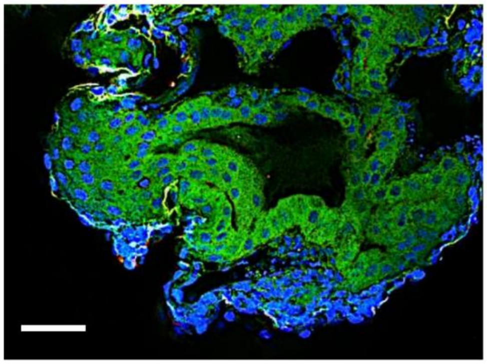

b

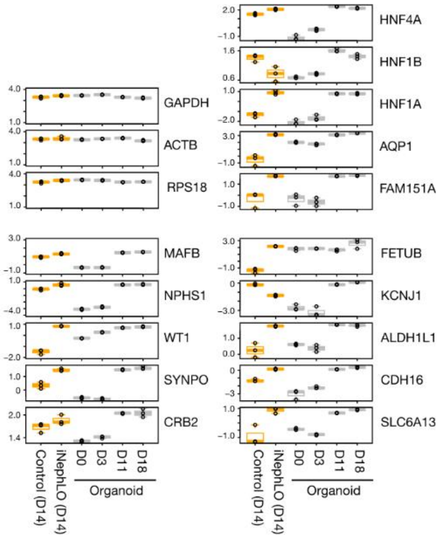

c

Sample	Control (D14) rep1	Control (D14) rep2	Control (D14) rep3	iNephLO (D14) rep1	iNephLO (D14) rep2	iNephLO (D14) rep3	Organoid (D0) rep1	Organoid (D0) rep2	Organoid (D0) rep3	Organoid (D3) rep1	Organoid (D3) rep2	Organoid (D3) rep3	Organoid (D11) rep1	Organoid (D11) rep2	Organoid (D11) rep3	Organoid (D18) rep1	Organoid (D18) rep2	Organoid (D18) rep3
GAPDH	3.178	3.316	3.348	3.496	3.419	3.393	3.452	3.478	3.422	3.525	3.536	3.498	3.314	3.304	3.292	3.280	3.183	3.211
ACTB	3.203	3.337	3.384	3.578	3.293	3.323	3.267	3.315	3.260	3.344	3.368	3.349	3.416	3.423	3.375	3.245	3.135	3.167
RPP40	0.586	0.848	0.757	0.695	0.817	0.806	0.853	0.797	0.917	0.913	0.936	0.990	0.760	0.734	0.748	0.694	0.766	0.651
RPS18	3.134	3.310	3.250	3.425	3.410	3.357	3.485	3.454	3.424	3.415	3.449	3.379	3.256	3.227	3.252	3.274	3.283	3.277
MAFB	0.835	0.951	0.995	1.282	1.266	1.401	-0.384	-0.325	-0.333	-0.343	-0.349	-0.302	1.445	1.463	1.447	1.553	1.523	1.529
NPHS1	-0.031	-0.255	-0.007	0.827	0.441	0.550	-3.194	-3.013	-3.108	-2.801	-2.836	-2.666	0.587	0.638	0.584	0.707	0.573	0.625
WT1	-1.373	-1.398	-1.736	0.819	0.868	0.816	-0.261	-0.283	-0.262	0.279	0.252	0.292	0.689	0.695	0.665	0.826	0.760	0.757
SYNPO	0.101	0.342	0.586	1.595	1.462	1.424	-0.587	-0.524	-0.643	-0.712	-0.601	-0.683	1.479	1.545	1.499	1.745	1.574	1.605
CRB2	1.526	1.724	1.757	2.007	1.763	1.811	1.292	1.333	1.314	1.424	1.380	1.432	2.047	2.049	2.030	2.164	1.945	2.042
ACTN4	2.299	2.403	2.458	2.707	2.517	2.514	2.386	2.409	2.379	2.207	2.204	2.222	2.560	2.587	2.553	2.859	2.732	2.778
ITGB5	2.036	2.185	2.199	2.071	2.038	1.996	2.158	2.168	2.110	1.991	1.993	1.956	1.949	1.953	1.949	2.250	2.109	2.134
GALNT10	1.520	1.795	1.701	2.128	2.147	2.169	2.061	2.059	2.031	1.848	1.856	1.833	1.963	1.993	1.946	2.108	1.992	2.034
EHD3	1.238	1.403	1.394	1.500	1.354	1.394	1.507	1.530	1.446	1.644	1.644	1.642	1.335	1.325	1.283	1.267	1.283	1.169
CD2AP	1.522	1.697	1.788	1.372	1.630	1.683	1.463	1.455	1.499	1.344	1.366	1.395	1.825	1.773	1.829	1.994	2.183	2.127
SH2D4A	0.718	0.465	0.883	-0.183	0.659	0.893	0.891	0.884	0.789	1.096	1.098	1.037	0.510	0.559	0.521	0.538	0.529	0.538
TJP1	1.929	2.207	2.192	2.044	2.102	2.148	1.835	1.818	1.838	1.855	1.835	1.837	2.291	2.374	2.337	2.484	2.456	2.452
LAMB2	1.927	1.972	1.973	2.439	2.256	2.352	1.515	1.564	1.519	1.748	1.752	1.772	2.431	2.520	2.435	2.545	2.333	2.438
ITGA8	1.164	1.407	1.394	1.441	1.592	1.732	1.802	1.761	1.787	1.684	1.634	1.672	0.601	0.615	0.620	0.989	1.004	0.977
DDN	-0.371	0.347	-0.008	-0.212	-1.378	-0.292	-2.845	-2.607	-2.539	-2.717	-2.353	-2.260	0.058	0.077	0.049	0.691	0.586	0.577
LMO7	0.824	1.138	1.007	1.484	1.585	1.537	0.542	0.649	0.570	0.414	0.470	0.534	1.600	1.644	1.627	2.046	2.046	2.026
CLDN5	-1.704	0.242	-1.146	1.697	1.482	1.315	-1.714	-1.174	-1.203	0.562	0.420	0.507	1.877	1.852	1.822	1.463	1.273	1.351
NID2	1.195	1.709	1.114	2.644	2.512	2.598	2.271	2.283	2.244	1.784	1.801	1.788	2.259	2.310	2.278	2.417	2.323	2.398
HNF4A	1.400	1.636	1.518	2.158	1.986	2.061	-0.846	-1.339	-1.318	-0.115	-0.229	-0.386	2.396	2.422	2.348	2.240	2.144	2.194
HNF1B	1.216	1.443	1.413	1.058	0.557	0.828	0.682	0.669	0.730	0.786	0.819	0.846	1.585	1.676	1.578	1.487	1.320	1.408
HNF1A	-1.192	-1.243	-1.583	1.089	0.710	0.872	-2.234	-1.816	-2.231	-1.323	-1.835	-1.770	0.785	0.820	0.739	0.862	0.711	0.773
AQP1	-1.446	0.119	-0.296	3.220	3.123	3.100	1.930	2.009	2.122	1.811	1.579	1.776	3.126	3.111	3.052	3.399	3.342	3.372
FAM151A	-1.167	0.066	0.032	1.782	1.715	1.723	-0.181	0.058	-0.955	-0.228	-0.974	-0.615	1.730	1.716	1.722	1.806	1.745	1.823
SLC22A5	0.538	0.804	0.829	1.364	1.446	1.384	0.314	0.423	0.425	0.425	0.505	0.420	1.469	1.430	1.426	1.556	1.505	1.568
SLC22A8	-1.432	-1.221	-1.304	-1.261	-0.213	0.248	-2.903	-2.785	-3.076	-2.857	-2.541	-2.714	-0.487	-0.562	-0.544	-0.950	-0.875	-0.836
SLC22A2	-0.283	-0.189	0.003	-0.202	-0.244	0.196	-0.700	-0.462	-1.277	-1.311	-1.590	-1.399	0.194	0.418	-0.032	0.534	0.776	0.911
SLC36A2	-1.557	-1.377	-0.306	0.915	0.593	0.675	-0.119	0.159	-0.118	0.027	-0.191	0.162	0.471	0.917	0.768	1.209	1.188	1.382
FETUB	-1.261	-1.112	-1.726	2.133	2.163	2.197	1.897	1.519	1.853	1.815	1.850	1.855	1.426	1.661	1.693	1.814	3.111	2.782
SLC12A1	-1.117	-0.260	-1.660	-1.224	-1.125	-1.763	-3.886	-3.456	-3.523	-2.924	-2.996	-3.036	-0.874	-0.844	-0.817	-0.520	-0.468	-0.498
KCNJ1	-0.061	-0.226	-0.194	-1.352	-1.280	-1.470	-2.842	-2.903	-2.332	-3.523	-3.456	-2.559	-0.181	-0.193	-0.134	0.106	0.086	0.074
ALDH1L1	0.249	0.704	-0.194	1.788	1.772	1.727	0.560	0.665	0.618	0.584	0.394	0.157	1.845	1.858	1.879	1.839	1.730	1.833
CDH16	-1.396	-1.108	-1.392	0.139	0.048	0.197	-2.670	-3.745	-2.759	-2.062	-2.273	-2.288	0.113	0.103	0.074	0.459	0.280	0.336
SLC4A1	-1.226	-1.330	-1.506	-1.100	-0.215	-1.681	-1.381	-2.465	-2.116	-1.428	-0.764	-1.398	-1.186	-1.518	-1.189	-0.970	-0.247	-0.321
SLC12A3	-1.409	-1.177	-1.557	-1.238	-1.158	-0.295	-1.559	-1.892	-1.941	-1.189	-1.224	-1.160	-0.772	-1.280	-0.775	-1.566	-0.164	0.077
CALB1	0.681	0.855	0.956	-0.201	-1.134	0.082	0.500	0.739	-0.301	0.453	-0.163	-0.414	0.871	-0.419	0.391	1.212	1.537	1.208
ATP6V0D2	0.117	0.456	0.338	0.102	0.837	0.199	-0.850	-0.318	-0.739	-0.303	0.140	0.028	0.784	0.929	0.810	0.376	0.577	1.026
SLC6A13	-1.301	-1.268	-0.138	0.649	0.968	0.975	-0.482	-0.450	-0.351	-0.798	-0.777	-0.851	0.692	0.681	0.675	0.942	0.866	0.914
PTH1R	1.479	1.496	1.660	1.188	1.157	1.348	-0.128	-0.262	-0.333	0.471	0.354	0.457	1.710	1.696	1.694	2.272	2.125	2.146
EMX1	-1.268	-1.272	-0.233	-1.206	0.159	-1.409	-2.759	-2.783	-2.796	-2.991	-2.955	-2.365	-0.851	-0.882	-0.823	-0.951	-0.816	-0.919
CLCNKA	-0.040	0.057	0.306	0.493	0.067	0.324	-1.286	-0.475	-0.504	-0.760	-0.795	-1.172	0.672	0.511	0.456	0.843	0.835	0.840

-3.00
-2.00
-1.00
0.00
1.00
2.00
3.00
normalized log10 (FPM)

	internal control				Podocytes																	
Sample	GAPDH	ACTB	RPP40	RPS18	MAFB	NPHS1	WT1	SYNPO	CRB2	ACTN4	ITGB5	GALNT10	EHD3	CD2AP	SH2D4A	TJP1	LAMB2	ITGA8	DDN	LMO7	CLDN5	NID2
Control (D14) rep1	3.18	3.20	0.59	3.13	0.84	-0.03	-1.37	0.10	1.53	2.30	2.04	1.52	1.24	1.52	0.72	1.93	1.93	1.16	-0.37	0.82	-1.70	1.20
Control (D14) rep2	3.32	3.34	0.85	3.31	0.95	-0.26	-1.40	0.34	1.72	2.40	2.18	1.80	1.40	1.70	0.46	2.21	1.97	1.41	0.35	1.14	0.24	1.71
Control (D14) rep3	3.35	3.38	0.76	3.25	0.99	-0.01	-1.74	0.59	1.76	2.46	2.20	1.70	1.39	1.79	0.88	2.19	1.97	1.39	-0.01	1.01	-1.15	1.11
iNephLO (D14) rep1	3.50	3.58	0.70	3.42	1.28	0.83	0.82	1.59	2.01	2.71	2.07	2.13	1.50	1.37	-0.18	2.04	2.44	1.44	-0.21	1.48	1.70	2.64
iNephLO (D14) rep2	3.42	3.29	0.82	3.41	1.27	0.44	0.87	1.46	1.76	2.52	2.04	2.15	1.35	1.63	0.66	2.10	2.26	1.59	-1.38	1.59	1.48	2.51
iNephLO (D14) rep3	3.39	3.32	0.81	3.36	1.40	0.55	0.82	1.42	1.81	2.51	2.00	2.17	1.39	1.68	0.89	2.15	2.35	1.73	-0.29	1.54	1.32	2.60
Organoid (D0) rep1	3.45	3.27	0.85	3.48	-0.38	-3.19	-0.26	-0.59	1.29	2.39	2.16	2.06	1.51	1.46	0.89	1.84	1.51	1.80	-2.84	0.54	-1.71	2.27
Organoid (D0) rep2	3.48	3.32	0.80	3.45	-0.32	-3.01	-0.28	-0.52	1.33	2.41	2.17	2.06	1.53	1.46	0.88	1.82	1.56	1.76	-2.61	0.65	-1.17	2.28
Organoid (D0) rep3	3.42	3.26	0.92	3.42	-0.33	-3.11	-0.26	-0.64	1.31	2.38	2.11	2.03	1.45	1.50	0.79	1.84	1.52	1.79	-2.54	0.57	-1.20	2.24
Organoid (D3) rep1	3.53	3.34	0.91	3.41	-0.34	-2.80	0.28	-0.71	1.42	2.21	1.99	1.85	1.64	1.34	1.10	1.85	1.75	1.68	-2.72	0.41	0.56	1.78
Organoid (D3) rep2	3.54	3.37	0.94	3.45	-0.35	-2.84	0.25	-0.60	1.38	2.20	1.99	1.86	1.64	1.37	1.10	1.84	1.75	1.63	-2.35	0.47	0.42	1.80
Organoid (D3) rep3	3.50	3.35	0.99	3.38	-0.30	-2.67	0.29	-0.68	1.43	2.22	1.96	1.83	1.64	1.39	1.04	1.84	1.77	1.67	-2.26	0.53	0.51	1.79
Organoid (D11) rep1	3.31	3.42	0.76	3.26	1.44	0.59	0.69	1.48	2.05	2.56	1.95	1.96	1.34	1.82	0.51	2.29	2.43	0.60	0.06	1.60	1.88	2.26
Organoid (D11) rep2	3.30	3.42	0.73	3.23	1.46	0.64	0.70	1.55	2.05	2.59	1.95	1.99	1.33	1.77	0.56	2.37	2.52	0.62	0.08	1.64	1.85	2.31
Organoid (D11) rep3	3.29	3.38	0.75	3.25	1.45	0.58	0.67	1.50	2.03	2.55	1.95	1.95	1.28	1.83	0.52	2.34	2.44	0.62	0.05	1.63	1.82	2.28
Organoid (D18) rep1	3.28	3.25	0.69	3.27	1.55	0.71	0.83	1.75	2.16	2.86	2.25	2.11	1.27	1.99	0.54	2.48	2.54	0.99	0.69	2.05	1.46	2.42
Organoid (D18) rep2	3.18	3.13	0.77	3.28	1.52	0.57	0.76	1.57	1.94	2.73	2.11	1.99	1.28	2.18	0.53	2.46	2.33	1.00	0.59	2.05	1.27	2.32
Organoid (D18) rep3	3.21	3.17	0.65	3.28	1.53	0.63	0.76	1.61	2.04	2.78	2.13	2.03	1.17	2.13	0.54	2.45	2.44	0.98	0.58	2.03	1.35	2.40

-3.00
-2.00
-1.00
0.00
1.00
2.00
3.00
normalized log10 (FPM)

	Proximal tubules									Distal tubules												
Sample	HNF4A	HNF1B	HNF1A	AQP1	FAM151A	SLC22A5	SLC22A8	SLC22A2	SLC36A2	FETUB	SLC12A1	KCNJ1	ALDH1L1	CDH16	SLC4A1	SLC12A3	CALB1	ATP6V0D2	SLC6A13	PTH1R	EMX1	CLCNKA
Control (D14) rep1	1.40	1.22	-1.19	-1.45	-1.17	0.54	-1.43	-0.28	-1.56	-1.26	-1.12	-0.06	0.25	-1.40	-1.23	-1.41	0.68	0.12	-1.30	1.48	-1.27	-0.04
Control (D14) rep2	1.64	1.44	-1.24	0.12	0.07	0.80	-1.22	-0.19	-1.38	-1.11	-0.26	-0.23	0.70	-1.11	-1.33	-1.18	0.85	0.46	-1.27	1.50	-1.27	0.06
Control (D14) rep3	1.52	1.41	-1.58	-0.30	0.03	0.83	-1.30	0.00	-0.31	-1.73	-1.66	-0.19	-0.19	-1.39	-1.51	-1.56	0.96	0.34	-0.14	1.66	-0.23	0.31
iNephLO (D14) rep1	2.16	1.06	1.09	3.22	1.78	1.36	-1.26	-0.20	0.92	2.13	-1.22	-1.35	1.79	0.14	-1.10	-1.24	-0.20	0.10	0.65	1.19	-1.21	0.49
iNephLO (D14) rep2	1.99	0.56	0.71	3.12	1.72	1.45	-0.21	-0.24	0.59	2.16	-1.12	-1.28	1.77	0.05	-0.22	-1.16	-1.13	0.84	0.97	1.16	0.16	0.07
iNephLO (D14) rep3	2.06	0.83	0.87	3.10	1.72	1.38	0.25	0.20	0.68	2.20	-1.76	-1.47	1.73	0.20	-1.68	-0.29	0.08	0.20	0.97	1.35	-1.41	0.32
Organoid (D0) rep1	-0.85	0.68	-2.23	1.93	-0.18	0.31	-2.90	-0.70	-0.12	1.90	-3.89	-2.84	0.56	-2.67	-1.38	-1.56	0.50	-0.85	-0.48	-0.13	-2.76	-1.29
Organoid (D0) rep2	-1.34	0.67	-1.82	2.01	0.06	0.42	-2.79	-0.46	0.16	1.52	-3.46	-2.90	0.66	-3.74	-2.46	-1.89	0.74	-0.32	-0.45	-0.26	-2.78	-0.47
Organoid (D0) rep3	-1.32	0.73	-2.23	2.12	-0.95	0.42	-3.08	-1.28	-0.12	1.85	-3.52	-2.33	0.62	-2.76	-2.12	-1.94	-0.30	-0.74	-0.35	-0.33	-2.80	-0.50
Organoid (D3) rep1	-0.11	0.79	-1.32	1.81	-0.23	0.42	-2.86	-1.31	0.03	1.81	-2.92	-3.52	0.58	-2.06	-1.43	-1.19	0.45	-0.30	-0.80	0.47	-2.99	-0.76
Organoid (D3) rep2	-0.23	0.82	-1.83	1.58	-0.97	0.51	-2.54	-1.59	-0.19	1.85	-3.00	-3.46	0.39	-2.27	-0.76	-1.22	-0.16	0.14	-0.78	0.35	-2.95	-0.80
Organoid (D3) rep3	-0.39	0.85	-1.77	1.78	-0.62	0.42	-2.71	-1.40	0.16	1.86	-3.04	-2.56	0.16	-2.29	-1.40	-1.16	-0.41	0.03	-0.85	0.46	-2.36	-1.17
Organoid (D11) rep1	2.40	1.58	0.79	3.13	1.73	1.47	-0.49	0.19	0.47	1.43	-0.87	-0.18	1.85	0.11	-1.19	-0.77	0.87	0.78	0.69	1.71	-0.85	0.67
Organoid (D11) rep2	2.42	1.68	0.82	3.11	1.72	1.43	-0.56	0.42	0.92	1.66	-0.84	-0.19	1.86	0.10	-1.52	-1.28	-0.42	0.93	0.68	1.70	-0.88	0.51
Organoid (D11) rep3	2.35	1.58	0.74	3.05	1.72	1.43	-0.54	-0.03	0.77	1.69	-0.82	-0.13	1.88	0.07	-1.19	-0.77	0.39	0.81	0.68	1.69	-0.82	0.46
Organoid (D18) rep1	2.24	1.49	0.86	3.40	1.81	1.56	-0.95	0.53	1.21	1.81	-0.52	0.11	1.84	0.46	-0.97	-1.57	1.21	0.38	0.94	2.27	-0.95	0.84
Organoid (D18) rep2	2.14	1.32	0.71	3.34	1.75	1.51	-0.88	0.78	1.19	3.11	-0.47	0.09	1.73	0.28	-0.25	-0.16	1.54	0.58	0.87	2.13	-0.82	0.84
Organoid (D18) rep3	2.19	1.41	0.77	3.37	1.82	1.57	-0.84	0.91	1.38	2.78	-0.50	0.07	1.83	0.34	-0.32	0.08	1.21	1.03	0.91	2.15	-0.92	0.84

Supplementary Figure S5

(a) Immunocytochemistry for proximal tubule (LTL) that shows tubule-like structures on day 14. Scale bar, 50 µm. (b) Principal component analysis for other human organs, corresponding control cell organoids (shown as Control (D14)) and iNephLOs (shown as iNephLO (D14)), kidney organoids reported previously (GSE70101, shown as organoid (D0, D3, D11, and D18) viewed from different directions. (c) Direct comparison between corresponding control cell organoids (shown as Control (D14)) and iNephLOs (shown as iNephLO (D14)), kidney organoids reported previously (GSE70101, shown as organoid (D0, D3, D11, and D18). Segment-specific makers are normalized by internal controls (GAPDH, ACTB, PRP40, RPS18). ; Podocyte markers; MAFB, NPHS1, WT1, SYNPO, CRB2, ACTN4, ITGB5, GALNT10, EHD3, CD2AP, SH2D4A, TJP1, LAMB2, ITGA8, DDN, LMO7, CLDN5, and NID2; Proximal tubular markers; HNF4A, HNF1B, HNF1A, AQP1, FAM151A, SLC2A5, SLC22A8, SLC22A2, and SLC36A2; Distal tubular markers; FETUB, SLC12A1, KCNJ1, ALDH1L1, CDH16, SLC4A1, SLC12A3, CALB1, ATP6V0D2, SLC6A13, PTH1R, EMX1, and CLCNKA.

Supplementary video 1

The three-dimensional (3D) image stacks were further processed using Imaris 3D software. It shows multi-segmented kidney structures containing podocytes (PODXL), proximal tubules (LTL), and distal tubules (CDH1) by day 14.

Supplementary data 1

Principal component analysis of transcriptome data obtained from various human organs, corresponding control cell organoids (shown as Control (D14)) and iNephLOs (shown as iNephLO (D14)), kidney organoids reported previously (GSE70101, shown as organoid (D0, D3, D11, and D18).

Supplementary Table S1. List of gene accession numbers

Transcription factor	Accession Number
FIGLA	BC148798
PITX2	BC013998
ASCL1	NM_004316.3
TFAP2C	BC051829
HNF1A	EU446660.1
GATA3	BC006793.1
GATA1	AB463674
EMX2	AB463605

Supplementary Table S2. List of primary antibodies

Antibody	Source	Catalog or clone number	Dilution
AQP1	Santa cruz	H-55	1:100
CDH16	home made		1:50
PAX2	BioLegend	PRB-276P	1:500
LRP2	kindly provided by Dr. Michiko Sekine, Tokyo Metropolitan Institute of Medical Science		1:100
PAX8	Proteintech	10336-1-AP	1:500
LHX1	Developmental Studies Hybridoma Bank	4F2-c	1:50
SIX2	Proteintech	11562-1-AP	1:500
SALL1	R&D systems	PP-K9814-00	1:100
EMX2	Abnova	H00002018-M02	1:100
CCND1	Abcam	ab134175	1:50
CDH1	Abcam	ab11512	1:100
CDH6	Atlas antibodies	HPA007456	1:500
JAG1	Santa cruz	C-20	1:50
CDH2	Abcam	ab98952	1:100
PNA	Vector laboratories	RL-1071	1:200
PODXL	R&D systems	AF1658	1:500
BRN1	Santa cruz	C-17	1:100
LTL	Vector laboratories	B-1325	1:200
THP	Alfa aesar	J65429	1:150
KIM1	R&D systems	AF-1750	1:200
HNF1A	Abcam	ab96777	1:100

Supplementary Table S3. Primer sequences

Gene	Forward	Reverse
ACTB	CAATGTGGCCGAGGACTTTG	CATTCTCCTTAGAGAGAAGTGG
CDH16	GGTACAGGTCAAGGACATGGG	TGCGGGTATAGGACTTTGAGAT
AQP1	TCTCTGTAGCCCTTGGACACC	GCCAGGATGAAGTCGTAGATGAG
PAX2	GCAACCCCGCCTTACTAAT	AACTAGTGGCGGTCATAGGC
LRP2	TGTGATGCAGCCATCGAACT	TGCATTTGGGGAGGTCAGTC
POU5F1	TGGAGTTTGTGCCAGGGTTT	CTTCACCTTCCCTCCAACCA
PODXL	GCAGAGAGGGCAAGAGTAAAACTG	GAGTCATCTGTGTCCTCCATGCT
NPHS1	CGCAGGAGGAGGTGTCTTATTC	CGGGTTCCAGAGTGTCCAAG
PAX8	CCCCCTACTCCTCCTACAGC	GGCCTTGATGTGGAACTGTAA
LHX1	ATGCAACCTGACCGAGAAGT	CAGGTCGCTAGGGGAGATG
BRN1	TGCCCAAAAGGAAAGATGTC	CATTCTCCTCGCAACAGTCA
SIX2	AGGAAAGGGAGAACAACGAGAA	GGGCTGGATGATGAGTGGT
WT1	GGCAGCACAGTGTGTGAACT	CCAGGCACACCTGGTAGTTT
SNAI2	CTGCGGCAAGGCGTTTTCCAGA	CAGATGAGCCCTCAGATTTGAC
VIMENTIN	ACACCCTGCAATCTTTCAGACA	GATTCCACTTTGCGTTCAAGGT
HNF1B	AGAAGCGTGCCGCTCTGT	TGGTTGAATTGTCGGAGGATCT
HNF1A	CCCACCAAGCAGGTCTTCAC	AAGGTCTCGATGACGCTGTG
