# Supplementary material for: Induction of human pluripotent stem cells into kidney tissues by synthetic mRNAs encoding transcription factors
Source: Sci Rep. 2019 Jan 29;9:913. doi: 10.1038/s41598-018-37485-8 (PMC6351687; doi:10.1038/s41598-018-37485-8)
Supplement: Supplementary file 3 — Supplementary Data 1 [file 41598_2018_37485_MOESM3_ESM.zip › Supplementary Data 1.html]

 
RGL model


You must enable Javascript to view this page properly.

  
Drag mouse to rotate model. Use mouse wheel or middle button
to zoom it.

---

  
Object written from rgl 0.99.16 by writeWebGL.
